# Evidence-based usability design principles for medication alerting systems

**DOI:** 10.1186/s12911-018-0615-9

**Published:** 2018-07-24

**Authors:** Romaric Marcilly, Elske Ammenwerth, Erin Roehrer, Julie Niès, Marie-Catherine Beuscart-Zéphir

**Affiliations:** 1 Univ. Lille, INSERM, CHU Lille, CIC-IT / Evalab 1403 - Centre d’Investigation clinique, EA 2694, F-59000 Lille, France, Maison Régionale de la Recherche Clinique, 6 rue du professeur Laguesse, 59000 Lille, France; 2 Institute of Medical Informatics, UMIT – University for Health Sciences, Medical Informatics and Technology, 6060 Hall in Tirol, Austria; 3 eHealth Services Research Group, School of Engineering and ICT, University of Tasmania, Private Bag 87, Hobart, Tasmania 7001 Australia; 4 General Electric Healthcare Partners, 92772, Boulogne Billancourt cedex, France

**Keywords:** Human engineering, Usability, Alerting system, Decision support, Design

## Abstract

**Background:**

Usability flaws in medication alerting systems may have a negative impact on clinical use and patient safety. In order to prevent the release of alerting systems that contain such flaws, it is necessary to provide designers and evaluators with evidence-based usability design principles. The objective of the present study was to develop a comprehensive, structured list of evidence-based usability design principles for medication alerting systems.

**Methods:**

Nine sets of design principles for medication alerting systems were analyzed, summarized, and structured. We then matched the summarized principles with a list of usability flaws in order to determine the level of underlying evidence.

**Results:**

Fifty-eight principles were summarized from the literature and two additional principles were defined, so that each flaw was matched with a principle. We organized the 60 summarized usability design principles into 6 meta-principles, 38 principles, and 16 sub-principles. Only 15 principles were not matched with a usability flaw. The 6 meta-principles respectively covered the improvement of the signal-to-noise ratio, the support for collaborative working, the fit with a clinician’s workflow, the data display, the transparency of the alerting system, and the actionable tools to be provided within an alert.

**Conclusions:**

It is possible to develop an evidence-based, structured, comprehensive list of usability design principles that are specific to medication alerting systems and are illustrated by the corresponding usability flaws. This list represents an improvement over the current literature. Each principle is now associated with the best available evidence of its violation. This knowledge may help to improve the usability of medication alerting systems and, ultimately, decrease the harmful consequences of the systems’ usability flaws.

**Electronic supplementary material:**

The online version of this article (10.1186/s12911-018-0615-9) contains supplementary material, which is available to authorized users.

## Background

Medication alerting systems “provide real-time notification of errors, potential hazards or omissions” related to the prescription of medications, and thus help clinicians to make informed decisions (*nota bene*: in the present report, a “clinician” is defined as any healthcare professional who interacts with the patient; the term therefore encompasses physicians, nurses and pharmacists) [1]. These promising technologies can change prescribers’ behavior by helping them avoid errors [2] and, ultimately, can improve the quality of the medication management process [3]. Nonetheless, the design and the implementation of these tools may introduce negative, unforeseen side effects: poor integration into the clinical workflow [4], acceptance issues, and decreased safety and quality of care, for example [5]. Some of these issues are related to the usability of the alerting systems [6]; they are caused by defects in the design of the system, i.e. usability flaws. For instance, alerts may be poorly integrated into the workflow and may appear too late in the decision-making process – rendering the alerting system useless [7, 8]. In other cases, the content of the alert is either incomplete or not visible enough to adequately support a clinician’s decision making – leading to incorrect clinical decisions [9]. This lack of information also increases the clinician’s cognitive load [10]. Alerts may be poorly written or explained - causing misunderstandings or at least creating difficulties in understanding them. These cognitive issues may also lead to incorrect clinical decisions [11–13]. In summary, these and other usability flaws in the alerting system may have severe consequences, such as rejection of the alerting system, and incorrect clinical decisions. Therefore, the usability of an alerting system warrants special scrutiny, with a view to avoiding usability-induced use errors at least.

To prevent the usability of alerting systems from introducing errors, usability activities (e.g. design specifications and prototype evaluation) must be undertaken during the technology development process [14]. The implementation of those activities requires a sound knowledge of good usability design principles (also known as usability heuristics and usability criteria). Violation of those principles may generate usability flaws in the technology. With a view to helping companies to avoid the release of medication alerting systems that contain unintentional violations of these principles, it is necessary to provide designers and evaluators with easy access to relevant, illustrated usability design principles and to convince them of the value of applying these principles to design decisions. In summary, designers and evaluators of medication alerting systems need to access evidence-based usability design principles, i.e. usability design principles that have proven their value in practice [15]. As far as we know, the present study is the first to have provided evidence-based usability design principles for medication alerting systems.

Putting together a body of evidence relies on the accumulation of results that demonstrate the positive value of applying design principles. Unfortunately, publications in the field of usability evaluation tend to report only negative results, i.e. instances of usability flaws. This reporting bias prevents the collection of evidence to show that applying principles is beneficial. Hence, although it is not yet possible to demonstrate the positive value of applying usability design principles, it is still possible to demonstrate the negative consequences of violating them.

**Fig. 1:**
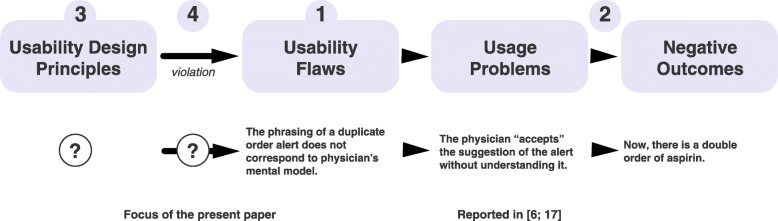
Top: a graphical representation of the evidence-based usability knowledge framework. The numbering refers to the four steps, as described in the text. The question marks refer to the steps tackled in the present study. Bottom: an instance of the cause-consequence chain linking a usability flaw, a usage problem and a negative outcome (adapted from [27])

In previous research, we started to develop a usability knowledge framework (Fig. 1; [16]). We have used this framework to gather evidence-based usability design principles for medication alerting systems. In a first step, we performed a systematic review of the literature to identify the usability flaws in medication alerting systems used in hospital and/or primary care (active or passive alerts, and use as a standalone system or integrated into a larger information system) [17]. In a second step, we searched for the consequences of these flaws on users (usage problems; e.g. alert fatigue and missed information) and on the work system (negative outcomes; e.g. a decrease in effectiveness, and patient safety issues), and linked them to their cause [6].

The third step involves identifying, summarizing, and organizing published design principles so as to avoid “reinventing” principles as far as possible. The fourth step (in line with previous work by Nielsen [18]) seeks to match usability flaws to the usability design principles that could fix them and thus obtain empirical illustrations of the principles’ violation. The present study tackled the third and fourth steps. The results will help to establishing evidence in support of these principles.

The present study had two objectives. Firstly, it sought to identify and organize literature reports of usability design principles for medication alerting systems in hospital or primary care settings into a comprehensive, structured list of design principles. Secondly, the study sought to match this list with the set of usability flaws identified in the systematic review [17], in order to assess the fit between known usability flaws and known existing design principles and thus illustrate violations of these principles.

## Methods

A two-step methodology was applied.

### Gathering and structuring usability design principles

We searched peer-reviewed journals and conference papers for published consensus sets of usability design principles for medication alerting systems (i.e. principles that experts in the field had agreed on). The “grey” literature was excluded because the quality of the information may vary. Hence, we searched PubMed, Scopus, and Ergonomics Abstracts databases for articles addressing both “medication alerting systems” and “usability” topics. With this goal in mind, we used the screening and eligibility assessment steps from our previous systematic review [17] to identify papers purposefully providing at least one usability design principle dedicated to medication alerting systems. We excluded system-specific papers providing recommendations on improving usability because these principles are not applicable to a broad range of systems. This task was updated on March 30th 2016. The literature search was intended to provide an overview of published sets of usability design principles for medication alerting systems, rather than being systematic and reproducible. The database search was completed by examining the investigators’ personal libraries and by screening the references of the selected publications. Two investigators (MCBZ and RM) decided on the final list of publications by consensus.

Once relevant publications had been identified, one investigator (RM) extracted all items referring to usability design principles from each publication. Next, the investigator grouped together principles with similar purposes and organized them hierarchically. A second investigator (MCBZ) independently crosschecked the hierarchical organization of the principles. Disagreements were solved by discussion until a consensus was reached. Lastly, the two investigators summarized the principles that had been grouped together.

### Matching usability design principles to known usability flaws

One investigator (RM) checked the list of usability flaws published in the on-line appendices of Marcilly et al. [17] against the structured list of usability design principles. A second investigator (MCBZ) crosschecked the results. Disagreements were discussed until a consensus was reached. The items referring to usability flaws were either descriptions of the technology’s defects observed during field studies or usability tests, answers to interviews/questionnaires, or users’ positive or negative comments about the characteristics of the technology collected during their interaction with the system. A usability flaw was matched to a given usability design principle if it was an instance of a violation of the said principle. Reciprocally, a usability design principle matched a usability flaw if the application of the principle stopped the flaw from occurring. If a flaw did not match any of the usability design principles, then we considered the possible extension (broadening) of an existing principle to other contexts so that it covered a wider range of flaws. If no principles could be extended to cover the flaw, we defined a new principle.

The matching process was intended to be as unequivocal as possible, i.e. one flaw matched one principle. However, if a given usability flaw violated several principles (e.g. at different levels of granularity), we matched that flaw to the most significantly violated principles (based on our experience). It should be noted that a given principle could be matched with several instances of the same flaw.

**Fig. 2:**
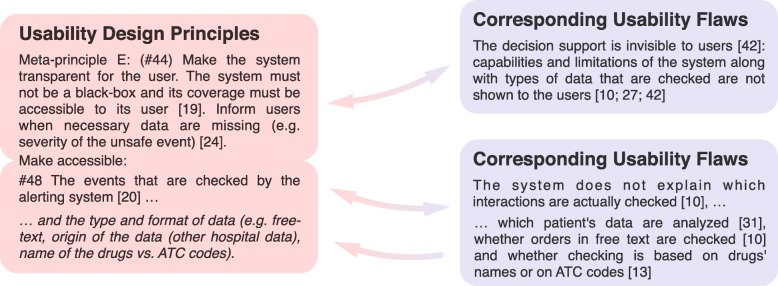
Illustration of the matching process, using meta-principle E (#44) and one of its sub-principles (#48). The usability design principles found in the literature were summarized and organized hierarchically (left). The usability flaws identified in the systematic review were collated by topic (right). Next, the correspondence between a given type of flaw and a given summarized principle was established based on the principle’s ability (if applied) to fix the usability flaw. This correspondence is represented by a double arrow. When a usability flaw could not be fixed by any of the design principles in the literature, we either extended an existing principle or created a new one (single arrow). The illustration presents an extension of principle #48 (in italics)

Figure 2 illustrates the matching process. Both investigators performed the descriptive analysis of the matches.

## Results

### Gathering and structuring usability design principles

We identified 9 publications on design principles dedicated to medication alerting systems (Table 1).

**Table 1:** Main characteristics of the publications on usability design principles

	First author	Year	Focus	Method used to provide the principles
[2]	Bates DW	2003	Design, implementation, monitoring	Lessons learned
[19]	Kuperman GJ	2007	Design, implementation	Expert consensus
[4]	Sittig DF	2008	Design, implementation, research	Lessons learned / expert consensus
[20]	Phansalkar S	2010	Usability	Targeted review
[21]	Pelayo S	2011	Usability	Targeted review & analysis of cognitive and collaborative tasks
[22]	Zachariah M	2011	Development of a usability evaluation instrument	Phansalkar et al.s’ review and feedback from a preliminary evaluation
[1]	Horsky J	2012	Usability	Targeted review
[23]	Horsky J	2013	Usability	Targeted review
[24]	Payne T	2015	Usability	Expert consensus

**Fig. 3:**
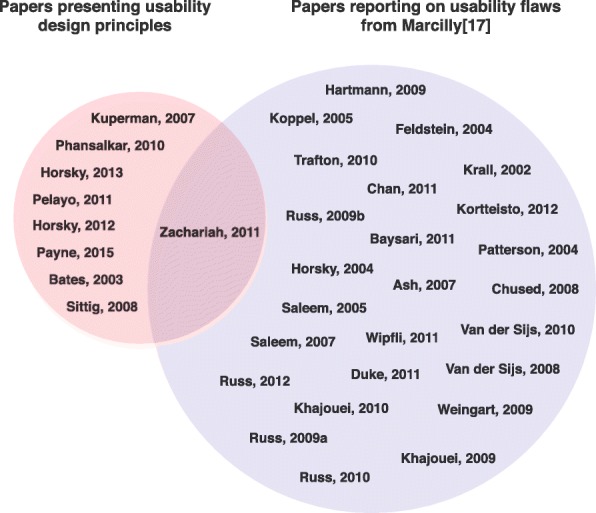
Sets of papers analyzed. Left: the set of papers analyzed to establish a structured list of usability design principles for medication alerting systems. Right: the set of papers analyzed to establish the list of usability flaws in medication alerting systems [17]

Figure 3 describes the sets of publications analyzed. One publication (Zachariah et al. [22]) was included in both sets; although this publication was an extension of another set of design principles described by Phansalkar et al. [20], it contained a few usability design principles not found in the original publication [20]. The publication also gave a list of usability flaws detected using heuristics. Despite the potential for self-matching bias, this publication was included because our objective was to obtain the most comprehensive possible list of design principles. Moreover, it was found that virtually all first authors of the set of usability design principles were co-authors of one or more studies included in the review of usability flaws (e.g.[1, 4, 20, 23]).

**Fig. 4:**
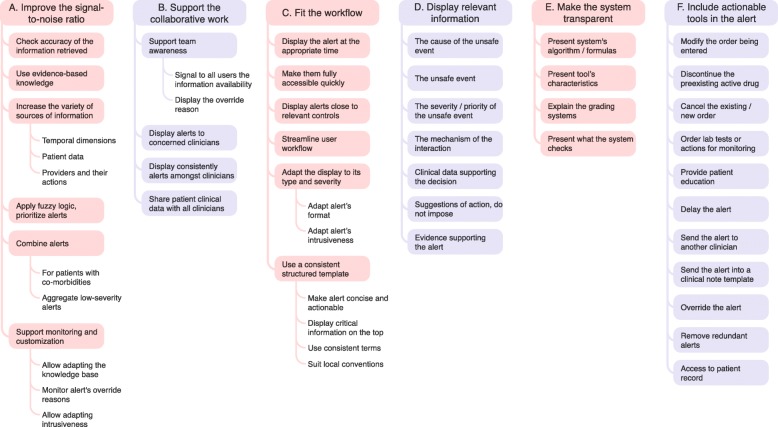
Hierarchical organization of the meta-principles, principles, and sub-principles specifically related to medication alerting systems. Meta-principles are displayed at the top in colored boxes. Principles are presented in the linked colored boxes. Sub-principles are presented in the border-free areas below the principles

A total of 345 items referring to usability design principles were extracted from the 9 publications (see Additional file 1: Appendix 1) and then organized. The level of agreement between the two investigators regarding the organization of the items was very high, with full agreement for 92.6% of the combinations, discussion needed for 6%, and disagreement for 1.3%. After a consensus meeting, the items were summarized into 58 principles. No significant inconsistencies between principles from different publications were noticed. The summarized principles displayed different granularity levels, and some were more tangible and precise than others; they could therefore be organized hierarchically into 6 meta-principles, 36 principles, and 16 sub-principles (Fig. 4, Table 2).

**Table 2:** Summarized usability design principles, and descriptions of the main corresponding flaws. Principles and sub-principles are presented respectively as first and second indents. Principles that have been added or extended to complete the matching process are given in italics. The oblique bars indicate the absence of corresponding flaws

Usability design principles	Summary of corresponding flaws
**Meta-principle A:** **#1 Improve the signal-to-noise ratio** by improving the sensitivity and the specificity of the alerting system in order to decrease the number of irrelevant alerts [1, 4, 19, 20, 23] *(e.g.,* system (non-medical) alerts, alerts with little evidence, low clinical relevance or redundant alerts, *alerts that require no action*).	There are too many alerts [8, 25–29] some are redundant [10, 25, 27, 30, 31] or irrelevant [11, 31], other do not need any action [29]. Potential events are over- or under-detected [10] due to sensitivity/specificity issues [32] or inappropriate triggering thresholds [11].
**#2 Check the accuracy of the information retrieved** from the CPOE (Computerized Physician Order Entry) /EHR (Electronic Health Record) [19], check whether they are outdated and / or reconciliated [1, 23]. **#3 Use adequate evidence-based alert knowledge base** [19]. It should be regularly up-dated/maintained [1, 2] (see #13).	Alerts are inconsistent with EHR data [10] especially with lists of patient’s actual medications [10, 12, 32, 33] or with patient’s diagnoses [34]. Medications interactions highlighted by the alerts are unknown in pharmaceutical reference books [27]. Knowledge supporting the alerts is not updated [34].
**#4 Increase the variety of the sources of information** used in the triggering model (e.g. several allergy bases [19]) and reconcile multiples entries [1]; when data are missing (degraded conditions), the system must continue to function [1].	/
**#5 Consider temporal dimensions:** interval between drugs’ administration [23]: distinguish “now”, “standing”, and “future” orders, evolution of the unsafe event: increase the severity of the unsafe event if it gets worse [21], time lab tests are overdue [1, 19], *interval between the appearance of the unsafe event and the administration of drugs.*	The alert is irrelevant because the adverse effect it presents happens too fast to be manageable [29]. The system does not distinguish orders specified as “now” and those specified as “future” or “standing” [31].
**#6 Consider patient clinical context **[1, 19, 23, 24]**:** besides the specific drug regimen(s) (e.g. dose, route, duration of therapy, sequence of initiating co-therapy, timing of co-administration), add patient and laboratory data into the expected interaction (e.g. age, gender, body weight, mitigating circumstances, predisposing risks factors, drug serum level, renal function, co-morbidity, and previous experiences). *Consider the point during patient’s stay at which the alert is presented*.	Medication order checking is not patient tailored [26, 35]: alerts may be valid but not applicable to patient clinical context [10, 31, 36]: e.g. pregnancy alerts for male patients and women of non-child-bearing age [32], no distinction between true allergies and side effects [10, 27]. An alert that is supposed to appear the last day of the stay (which is unforeseeable) appears every day [34].
**#7 Consider actions already taken by the provider** (e.g. dose adjustment) [21] **and provider-specific data **[1, 23] (e.g. clinical specialty: *some drugs may be used off-label,* others may be voluntarily duplicated triggering “duplicate orders” [19]).	Alerts appear while the corrective or monitoring actions have already been taken by the physicians [29]. Some corrective actions that are clinically relevant are not accepted by the system [36]. In some specialties, adverse events are intentional [29, 32] (e.g. psychiatry [32]); in other, drugs are used off-label (e.g. pediatrics [32]). Clinicians already know the alerts [25, 29].
**#8 Include fuzzy logic-based algorithms**, multi-attribute utility model and filters into the triggering model to change alerts’ activation when certain conditions apply [1, 19, 20]. Define appropriately thresholds to trigger the alerts and to prioritize the alerts according to the patient’s clinical context and the severity of the unsafe event [1, 4, 20, 23] (see #28).	Alerts triggering thresholds are too low [26]. Alerts are not ordered by severity level [22]. Non-significant, or low incidence, alerts are presented [11, 12, 29].
**#9 Combine alerts** [1]**:**	/
**#10 Recommendations must be combined in a consistent way for patients with co morbidities **[4]**.**	/
**#11 Aggregate low severity alerts in a single display** to be reviewed all at once at a convenient point in the workflow [1, 20, 23].	Alerts are not grouped according to their severity [22].
**#12 Support monitoring of the usage of alerts and their customization** [1, 2, 19, 23, 24]	/
**#13 Allow expert committees in each organization to adapt the knowledge-base and suggestions** for action to local practices and guidelines and to remove potential errors from the base. Customizations should persist across version upgrades [19, 23, 24].	Alerts are in conflicts with local and common practices [10, 27, 36].
**#14 Monitor alerts’ override reasons**: alerts frequently overridden and of little value should be considered for removal or for a change of their presentation format (e.g. intrusiveness) [1, 2, 19, 23, 24]. However, do not eliminate or turn off relevant alerts even for specialists [24].	/
**#15 Allow institutional flexibility in determining interruptive vs. non-interruptive alerts** [23, 24].	/
**Meta-principle B:** **#16 Support the collaborative work.** Advocate a team approach [24], make the alerting system a team player [21].	The system does not inform physicians whether pharmacists review their justification of override and / or find them useful [10].
**#17 Provide functions to support the team awareness of the alert management** [21, 24]: (see #56).	/
**#18 Signal the availability of information to all users **[21] (even to non-prescribing clinicians [24]).	/
**#19 Display override reasons** entered by a physician to nurses and pharmacists in order to allow them to understand the rationale for overriding [1, 19, 21, 23, 24].	Justifications and comments are displayed to no one [37]
**#20 Display alerts to concerned clinicians** and then to non-prescribing clinicians as a second check [24]. Redirect alerts that do not concern physicians to support staff [1].	Pharmacists receive alerts that concern physicians (e.g. drug interaction [38]). Physicians receive alerts related to drugs administration that concern nurses [11, 29]. Physiotherapists and nurses receive alerts related to the ordering of drugs while they do not prescribe [26].
**#21 Display consistently the basic alert content, i.e. the main elements of the alert, amongst all clinicians **[21, 24]. Nonetheless, the detailed presentation may differ based on clinicians’ expertise (e.g. pharmacists may need more pharmacological data) [1, 23], on their role (privileges, responsibilities) and on the context of use [24]. Details may be presented upon request [21].	The way data are displayed is not adequate for all clinicians’ types [10].
**#22 Share patient clinical information in the alert summary screen with all clinicians** (e.g. with pharmacists) [1, 4].	/
**Meta-principle C:** **#23 Fit the clinicians’ workflow and their mental model **[1, 2, 4, 20]**.**	Mental model implemented in the system does not fit users' one [12].
**#24 Display the alert at the appropriate time** during the decision making[1, 2, 24] or later during the medication management process [19].	Alerts appear out of the logical workflow [30], either too late in the ordering process [7, 8, 25, 31, 38, 39] or too early [7, 31].
**#25 Alerts must be displayed and fully accessible quickly**: screen transition time must be well under a second [1, 2], avoid scrolling and tabs [24].	Alerts appearance is delayed [12]: lags/down-times of 8 sec. [28] up to 15 sec. [10]. Clinicians must explore several parts of the alerts to get all relevant data [9]: they must scroll [10, 11, 22], explore several tabs [9, 40], or find information in tooltips [26] because short versions of alerts are not sufficiently informative [31].
**#26 Display alerts over the CPOE/EHR screen** in close proximity to the relevant controls and displays [20, 23].	Alerts are outside the region of the screen where clinicians are looking [26, 34].
**#27 Streamline users’ workflow in response to alerts** [22]. Make the resolution of alerts quick and easy (few steps) through screen operations [1, 22, 24]: cancel or reset alerts in response to the appropriate corrective action, do not require acknowledgment before a corrective action [20]. After the alert is resolved, resume the workflow [19] (see #49).	/
**#28 Adapt the display of the alert** to its type (medication alerts, system alerts) and its severity [19, 20, 23, 24].	Alerts of different severity levels and of different types are not distinguished [22].
**#29 Adapt alert’s format** (e.g. color, symbol) and location on the screen [20, 24]. More severe alerts must be placed within the focal region of the user’s visual field in order of importance while non severe alerts must be placed in side regions [1, 20, 23]. Distinguish system vs. medication alert messages [20].	Alerts are not distinguished by severity nor by type [10, 22, 32]. All alerts look the same [22]
**#30 Adapt alert’s intrusiveness **[1, 4, 19, 21, 23 24]. Interruptive alerts should be reserved for high severity warning and used judiciously: they should require an explicit response. Less important alerts must be displayed less intrusively (e.g. on-demand) as messages not requiring any actions. *Do not use pop-up alerts for system messages.*	High risk alerts are not seen when not intrusive [31]. On the contrary, low risk alerts that pop-up annoy users. Moreover, non-medication alerts are too intrusive [30] and contribute to desensitize the users [10, 27].
**#31 Use a consistent structured alert template across the various systems used by the clinicians** [23, 24].	Alerts combined but not structured cause visualization difficulties [10, 12, 27, 34]: users face difficulties to find specific data [11]. Thelack of guidance bother users and their understanding [30, 35, 41]. Useful ondemand information available in EHR is not available in the alerts [22].
**#32 Make the alert concise and actionable**; the description of the problem should be shorter than 10 words [1, 19, 24, 24]; labels of button must be concise too [1] (see #49).	Alerts contain too much text or extraneous information [10, 25, 30, 41].
**#33 Present the most critical information on the top-level of the alert**: the unsafe event, its causes and its severity [1, 23, 24]. **Then display on-demand (linked) information** on background and secondary considerations (contextual information, mechanism of interaction and evidence[19, 24]. The suggestion of action could be presented either at the top level or on-demand [20, 24] (see #36).	No data are highlighted within the alert [9, 10], the alert is on a paragraph form [41].
**#34 Use consistent terms**, phrases, classifications, colors and definitions (e.g. for the severity) [1, 21, 22].	/
**#35 Terminology and messages should suit local conventions** [23] and be understandable and non-ambiguous [1, 23].	The message conveyed by the alert is not understandable [10, 12, 40]: the text is ambiguous [41]. Icons used are misinterpreted [28, 41] as well as buttons labels [35].
**Meta-principle D:** **#36 Display relevant data within the alert** [1, 20, 23, 24] (see #33). For the relevant tools to propose, see meta-principles F.	/
**#37 The cause of the unsafe event** *and its characteristics (e.g. dose)* [1, 19, 20, 23]. Use the medication name as ordered as well as generic drug names when identifying the interaction [24], do not focus on pharmacological / therapeutic classes [24].	Alert does not provide information on why it is triggered [10, 12, 27].
**#38 The unsafe event** (potential or currently happening) [19, 20, 23, 24]. Do not use generic term (e.g. risk), prefer concrete description [24]. Present the frequency or incidence of the unsafe event [24].	Alert does not inform on the unsafe event [9, 10, 12, 27].
**#39 The severity / priority of the unsafe event **[1, 20, 23, 24]: use color code and a signal word to inform on the severity [20].	Alert does not inform on the severity of the unsafe event [9, 10, 27].
**#40 The mechanism of the interaction** (possibly by embedded links) [23, 24].	Alert does not provide information on how the causes of the unsafe event conduct to the unsafe event [9].
**#41 Relevant data supporting the decision-making process** and the suggestions of action [1, 4, 23, 24]: e.g. contextual information, modifying and predisposing factors (e.g. co-morbidity or lab-values). Provide a link to a summary of patient clinical data [23]. *Display data necessary to interpret values provided (e.g. thresholds).*	The alert does not provide essential patient information for the prescriber [10]. User can link to outside sources of information from elsewhere in the system, but there is no link within the alert [22]. Even when alerts provide patient biological results, the thresholds to interpret them are not presented [9].
**#42 Suggest, do not impose.** Make the system a clinician’s partner [21]: provide clinically appropriate suggestions of action for mitigating the potential harm, do not impose [1, 19, 20, 23, 24]. Present possible ancillary orders such as monitoring/surveillance actions, drug alternative (incl. Dose and frequency) and / or order modification or cancellation [1, 2, 23, 24]. Make the suggestions actionable [24] (see meta-principle F). In case of multiple suggestions, prioritize them and present their conditions of application [24]. Justify suggestions [21, 24]: check locally suggestions [24] and include link to institution-specific guidelines [19], make consensual suggestions [1, 19]. Monitor whether or not users followed through with the suggested action they started; if users fail notify them they did not finish the action they started [22].	Alerts do not provide suggestions of action [10, 22, 29, 41] nor alternative treatment options [9]. Alerts provide erroneous suggestions of action [35].
**#43 Evidence supporting the alert** (incl. Strength and source) using symbols/letter/numbers [24]. Include a link to a more complete documentation (monograph, evidence, extended information, context) [1, 2, 19, 23, 24].	Alerts do not provide existing evidence or the evidence that supports the alert is poor and contradict clinicians’ knowledge [10, 27, 29]. The alert does not present evidence references [9].
**Meta-principle E:** **#44 Make the system transparent for the user.** The system must not be a black box and its coverage must be accessible to its user [19]. Inform users when necessary data are missing (e.g. severity of the unsafe event) [24]. Make accessible:	The decision support is invisible to users [42]: capabilities and limitations of the system along with types of data that are checked are not shown to the users [10, 27, 42].
**#45 The alerting algorithm / logic / formulas implemented within the system** [1, 20, 23].	No alerts are appearing after ordering medications although clinicians expect one to come up for a patient [12]. The calculation formulas that the system applies are not understood by clinicians [7].
***#46 A description of the characteristics of the tools included in the alert (e.g. duration of activation).***	The system is not explicit about how to use and manage alerts effectively [31]: it does not make it clear that one can turn off some alerts [10] and for how much time [36].
**#47 Explanations on the grading systems**: levels of severity used by the alerting system (and their number by unsafe event) [20, 24], and explanations of their classification as unsafe events [20]).	The system does not explain the levels of severity that are used [22].
**#48 The events that are checked by the alerting system** [20] and *the type and format of data (e.g., free-text, origin of the data (other hospital data), name of drugs vs. Anatomical Therapeutic Chemical-ATC codes)*	The system does not explain which interactions are actually checked [10], which patients’ data are analyzed [31], whether orders in free text are checked [10] and whether checking is based on drugs’ names or on ATC codes [13].
**Meta-principle F:** **#49 Include actionable tools within the alert** to allow clinicians to take actions intuitively, easily and quickly [1, 2, 4, 19–24]; display those tools close to the suggestions they are related to [23] (see #27 and #32). The list of actionable tools should include:	There are dead ends in which clinicians face no reasonable options to proceed [28]; there are no useful actionable options [22].
**#50 Modify** the order being entered (or its dose) [1, 22–24]. For instance, propose formulary drugs lists [19] for formulary drug alerts. Allow ordering a drug suggested [23] or a new drug: in this case, clearly state that the existing drug will be discontinued if the new one is finalized [23], open a pre-populated ordering screen for the new drug [23].	The system does not guide users for switching a medication [41].
**#51 Discontinue** the preexisting active drug [22–24].	The system does not guide user for discontinuing a medication [41].
**#52 Cancel** the existing / new order [1, 22–24].	/
**#53 Order lab tests or actions** for monitoring as justified by the alert [1, 24].	/
**#54 Provide patient education** [24].	/
**#55 Delay the alert** for a predetermined amount of time (“snooze” function) [24], *allow users to get the alert again.*	Alerts cannot be pulled up later, hindering alert resolution [10, 36]. Moreover, it is not possible to get the alert again [11].
**#56 Send the alert to another clinician** [24].	The system does not support the transmission of alerts to others clinicians [28].
***#57 Send the alert into the clinical note template***	The system does not allow clinicians to send the alerts into a template for patient’s record [28, 41].
**#58 Override** the alert (meaning continue ordering, ignoring the alert) [22–24]. Most severe unsafe events must be more difficult to override (e.g. require a second confirmation, or even no possibility of overriding [23]) than less severe ones. Alerts must require the reason for override [24] (especially the most critical ones, optional otherwise [23]. Avoid text entries, propose a list of 3–4 (max 5 items) selectable coded reasons; reasons must be 1–2 word long [19, 23, 24].	The system does not provide appropriate options for justifying overrides [28]. The system is not explicit about the necessity to enter a justification [37]; moreover, free-text entries are not effective in the override justification logic [10] and entering data to justify overrides is seen as time burden [10, 36].
**#59 Allow providers to remove redundant alerts** for a patient who has previously tolerated a drugs combination (after more than one override) or when providers feel they have sufficient practice and knowledge about this alert or when the alert is outdated for a specific patient [1, 23].	The system does not provide users the possibility to remove irrelevant alerts that therefore continue to appear [28].
**#60 Allow users access easily patient’s record from the alert screen** to change erroneous data (e.g. allergy) or to add new data. Do not require entering additional data in the alert [1, 19].	The system asks the users to enter data in the alert and then in the patient record, leading to wasting time and double documentation [28].

Overall, the 9 publications contributed to different extents to the set of summarized principles: the contributions ranged from 12% [22] to 69% [24] (see Additional file 2: Appendix 2).

The level of support for each of the summarized principles (in terms of number of publications in which they were found) varied: one summarized principle was supported by all 9 papers (#49 “Include actionable tools within the alert”), “Suggest, do not impose” (#42) was supported by 8 publications, and 6 principles were found in 1 publication. When considering the overall meta-principles and their components (i.e. related principles and sub-principles), the level of support ranged from 5 publications for meta-principle E (“Make the system transparent for the user”) to 9 publications for meta-principle D (“Display relevant data within the alert”) and meta-principle F (“Include actionable tools within the alert”).

### Matching usability design principles with known usability flaws

The two investigators agreed well on the matching between usability flaws and usability design principles, with full agreement for 54.6% of matches, partial agreement for 31% (agreement on the main corresponding principle but a need to match the flaw with a second principle), discussion needed for 13.4%, and disagreement for 1%. Of the 58 principles, 34 directly matched at least one instance of a usability flaw, nine were broadened to cover a flaw, and 15 were not matched at all (see Additional file 3: Appendix 3). Two new principles were defined so that all flaws matched a principle (#46, provide “a description of the characteristics of the tools included in the alert” to users, and #57, include a “send the alert into the clinical note template” function in the alert).

After the addition of the 2 new principles, the final set comprised 60 summarized usability design principles: 6 meta-principles, 38 principles, and 16 sub-principles. The 6 meta-principles were as follows:

A. **Improve the system’s signal-to-noise ratio**, in order to decrease the frequency of over-alerting. In addition to the drugs ordered, the alert strategy should take into account parameters such as the patient’s clinical context or the clinician’s specialty. Moreover, the system must provide tools to customize the knowledge implemented within it and to monitor alert overrides.
B. **Support collaborative work, advocate a team approach, and make the system a team player**. The alerting system must encourage collaboration between the healthcare professional managing medications (e.g. physicians, pharmacists and nurses). Overall, alerts must deliver the same information to all clinicians, even if additional supplementary data can be presented as a function of the healthcare professional’s role. The alerting system must help clinicians to understand how other healthcare professionals have already managed the alert.
C. **Fit with clinicians’ workflow and their mental model**. The alerting system must comply with clinicians’ needs and tasks. Alerts must be presented at the right moment in the decision-making process. Only the most severe alerts must interrupt the users; other alerts must be displayed more discreetly. Alerts must be concise, understandable and consistently structured so that users can easily find the relevant data. Once the alert has been satisfied, the clinicians must be able to resume their tasks easily.
D. **Display relevant data within the alert**. The system must provide clinicians with the information needed to make informed decisions. This includes the cause of the unsafe event (the medications involved), the description of the unsafe event, the severity/priority of the event, the mechanism of the interaction, the patient’s clinical context, and evidence supporting the alert. Lastly, the system must suggest – but not impose – a means of remedying or monitoring the unsafe event.
E. **Make the system transparent for the user**. The alerting system must help clinicians to understand what the system can and cannot do and how it works, in order to prevent erroneous interpretation of its behavior. The user must have access to (i) the types of data that are checked, (ii) the formulas and rules applied, (iii) the list of the unsafe events that are targeted, and (iv) a description of the alerts’ levels of severity.
F. **Include actionable tools within the alert**. The alert must provide several tools that help clinicians to easily and quickly translate their alert-informed clinical decision into actions: for example, buttons to modify/cancel/discontinue an order or override the alert, to order actions for monitoring an event, and to provide patient education. Other tools are recommended for managing the alert: pulling up the alert at a later time, sending the alert into a clinical note, removing the alert for a patient, and gaining access to the patient’s medical records.

The final list of summarized usability design principles is given in Fig. 4. Table 2 provides a detailed version of the principles and corresponding flaws.

## Discussion

### Answers to study questions

The present study sought primarily to provide a specific, comprehensive, structured list of usability design principles for the medication alerting systems implemented in hospital or primary care settings. The secondary objective was to pair this list with the set of documented usability flaws, assess the match between the usability flaws that are known and the existing design principles, obtain illustrations of the existing violations of the principles, and present evidence that not applying usability design principles may be detrimental.

A total of 60 specific usability design principles for medication alerting systems were identified and organized hierarchically around 6 meta-principles: (A) improve the signal-to-noise ratio, (B) support collaborative work, (C) fit the clinicians’ workflow and their mental model, (D) display relevant data within the alert, (E) make the system transparent for the user, and (F) include actionable tools within the alert. The 9 analyzed publications contributed to this list to different extents; we consider that the collation of several sets of usability design principles found in the literature expands the variety of topics represented in each individual set.

The match between the summarized usability design principles and the list of documented usability flaws was quite good: 34 principles were directly matched, and the context of application was extended for 9 principles. Nonetheless, 15 principles did not match any of the documented usability flaws. In view of the hierarchical organization of the principles, some principles are also not matched because their meta-principle or one or more of their sub-principles are matched - thus artificially reducing the quality of the match. We also identified limited gaps in the principles found in the literature; two new principles had to be created.

From a qualitative point of view, a few instances of usability flaws appear to contradict the corresponding usability design principles. For instance, some principles recommend including non-prescribers (e.g. pharmacists and nurses) in the alert management process, in order to promote collaboration between healthcare professionals (e.g. #20). However, it has been reported that nurses are annoyed by medication alerts that interrupt their work [26]. The balance between promoting collaboration between healthcare professionals and not disrupting non-prescribers’ tasks is delicate. Overall, instances of usability flaws must be used so that the corresponding design principles are not taken too literally.

### Study strengths

The results of the present study represent an improvement with respect to the current literature. We did not change the principles extracted from the literature. By combining and summarizing the extracted principles, they are now clearly identified, listed, and organized hierarchically into a comprehensive, consistent, and structured hierarchy. Furthermore, the process of matching the principles to the usability flaws allows one to identifying evidence to show that not applying these principles has negative consequences. Each principle is now associated with the best available evidence of its violation. As far as we know, the present study is the first to have drawn up this type of list.

In addition to providing evidence, the matching process also provided concrete illustrations of violations of usability design principles. The illustrations may help people designing and evaluating alerting systems to identify the “usability mistakes” that should not be made or to catch these mistakes during the evaluation phases. In fact, the illustrations provide a clearer understanding of the design principles to be applied.

### Study limitations

The retrieval of the usability design principles might have biased the representativeness of the principles and the flaws. We considered only publications reporting general sets of design principles, rather than evaluations giving system-specific usability recommendations. Grey literature was excluded. Moreover, most of the analysis was performed by one investigator, with a second investigator independently crosschecking the results. Together, these biases might have caused us to miss a few relevant principles. Consequently, the principles that we extended or created in the present study may have already been described in other publications (e.g. as system-specific recommendations on usability). Likewise, some usability flaws might have been missed during the systematic review [17] due to publication and reporting biases: it might have been possible to match principles not matched in the present study with usability flaws documented outside our review [17]. Despite these limitations, the match between the usability design principles and the usability flaws was quite good and ensured that the principles and flaws retrieved were representative. This good level of matching might be due (at least in part) to the inclusion of Zachariah’s publication [22] and reports written by closely linked authors in both sets of publications (i.e. the set used to establish the list of principles and the set used to establish the list of flaws, e.g. [4, 20, 23]). In the present study, the risk of self-matching bias was considered to be acceptable because our objective was to obtain the most comprehensive possible list of design principles and corresponding flaws. On the contrary, not including a publication in one set because its authors had also worked on a publication included in the other set could have led us to ignore relevant usability design principles and/or usability flaws.

The frequency of appearance of the design principles was analyzed in order to establish the level of support for the design principles (i.e. the number of publications they were found in). However, we do not interpret this number as an indicator of which principles should be prioritized. Firstly, reporting and publishing biases and differences in the focus of the publications analyzed may have biased the frequency of appearance. Even without these biases, prioritizing the principles would imply that we are able to predict the severity of the consequences of the related usability flaws. However, the severity depends on many other factors, such as the system’s other features and other flaws, and the context of use. This is one reason why most sets of design principles - whether developed for interactive systems (e.g. Nielsen’s [43] and Scapin’s [44] sets) or for a specific type of technology (e.g. the ones included in our analysis) - do not prioritize principles. Design principles can be prioritized by a person who is aware of the alerting system’s characteristics and context of use.

In the present study, we addressed the evidence in favor of usability design principles by examining the violation of these principles. Evidence to suggest that applying design principles is beneficial has not yet been considered, due to reporting bias in the literature. Even though our present evidence is not based on instances of successful design, it may be convincing enough to persuade designers to apply usability design principles. Once researchers have begun to report on the positive usability characteristics of medication alerting system, the present analysis will have to be updated.

### The significance of the present results for a user interacting with a medication alerting system

**Fig. 5:**
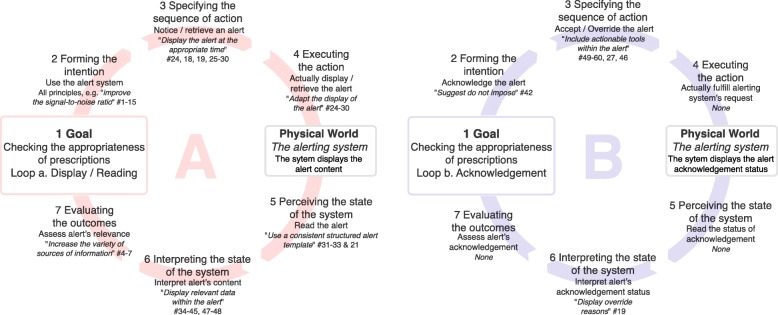
Norman’s “seven stages of action” model, as applied to the interaction with a medication alerting system. Top: Loop a (on the left; “display/read” the alert) represents the core interaction. Loop b (“acknowledgement”) represents a second order interaction: not all alerting systems require alerts to be acknowledged. Usability design principles that could (if applied) improve the quality of the interaction are linked to the corresponding stage of the model, when possible

Usability design principles are related to various components of the alerting system: the triggering model, the knowledge implemented, the cognitive model implemented in the system, the information displayed, and the tools proposed within the alert. Applying these usability design principles might improve the clinician-alerting system interaction and the collaboration between clinicians. According to Norman’s “seven stages of action” model [45], the user’s interaction with a system encompasses two stages: the action stage translates a goal into an action sequence, and the evaluation stage compares the changes perceived in the world with the initial goal of the action (see Fig. 5). A clinician interacts with an alerting system in order to check the appropriateness of the prescriptions (step 1). Two “action and evaluation loops” may then be described. The main loop is “display/read” the alert. The second “acknowledgement” loop depends on the alerting system model; in some models, acknowledgment is not required.

For the “display/read” loop (loop a, Fig. 5, left), improving the overall usability of the alerting system by applying the whole set of design principles may facilitate the interaction and increase the clinician’s intention to use the alerting system (step 2). More specifically, the whole “improving the signal-to-noise ratio” meta-principle may help to improve the relevance of the alerts, decrease alert fatigue, and thus increase the clinician’s will to use the alerting system. In step 3, principles such as “signal the availability of information to all users” (#18) and “display the alert at the appropriate time” (#24) could make it easier to notice and retrieve alerts. In step 4, applying the “fit the clinician’s workflow” meta-principle may help the clinician to display the alerts. Once alerts are displayed, clinicians have to read and interpret them (in steps 5 and 6). Applying the “use a consistent structured alert template”, “display relevant data”, and “make the system transparent” principles (#31, #36, and #44, respectively) may make the alerts more readable and help the clinicians to interpret them. Lastly, “extend[ing] the sources of information used in the triggering model” (#4) may make it easier for clinicians to assess the alerts’ relevance (step 7).

Once alerts are interpreted, physicians may have to acknowledge them (loop b, Fig. 5, right). Applying the “suggest - do not impose” principle (#42) may increase the probability with which a clinician acknowledges the alert and perform corrective actions (step 2). Next, “includ[ing] actionable tools within the alerts” (#49) may make it easier and quicker to specify and execute corrective actions (e.g. modify the order; step 3). If a physician overrides an alert and enters the reason why, “display[ing] override reason” (#19) might help other clinicians to interpret the alert’s acknowledgement status (step 6) and decide whether or not the alert has been properly assessed.

In summary, applying this set of usability design principles might improve both the action and evaluation stages of a user’s interaction with the alerting system - mainly in the “display/read” loop but also in the “acknowledgement” loop. Some principles go beyond Norman’s model, which relates to an individual’s interaction with the alerting system and not interactions between clinicians or the clinicians’ workflow. Adhering to the “fit the clinicians’ workflow” meta-principle might decrease the risk of rejection. Moreover, if the “support collaborative work” meta-principle were to be applied, the alerting system could truly help clinicians to gain the same mental representation of the prescription being checked; this would help them to coordinate their actions and improve patient safety.

### Generalizability of the study

The list of usability flaws used in the matching process might increase over time, depending on whether new publications report usability flaws. Moreover, technology evolves rapidly, and the related principles might change accordingly. For instance, the principles presented here are formulated for medication alerting systems implemented on laptop and/or desktop computers. However, as mobile health technologies are refined and expanded, alerting systems will be progressively installed on mobile devices. This might modify the applicability of the usability design principles listed here. It will therefore be essential to update this work regularly and take account of the latest trends and developments. However, the maintenance of this knowledge may be time-consuming, and represents a challenge for human factors specialists in the field of medical informatics. Manufacturers should be associated with this process.

Some design principles insist on the need for promoting collaboration between clinicians (#16) but ignore the key person in the medication management process - the patient. Only principle #54 mentions the patient (being able to “provide patient education”). However, as for other information technologies, the implementation of an alerting system changes the nature of the patient-clinician interaction [46]. It is important to ensure that poor usability has not damaged the patient-clinician interaction. On the contrary, increased usability should underpin patient-clinician discussion, empower the patient [47], and ensure that care remains patient-centered. The current literature on the usability of medication alerting systems does not consider the patient as a stakeholder in medication management. Future research on the usability of medication alerting system should integrate patients as stakeholders in medication management, so as to adapt or extend usability design principles to their specific features.

Although the structured design principles target only medication alerting systems implemented in hospital or primary care settings, some principles may be applied to other kinds of alerting systems. For instance, part of the “fit the clinicians’ workflow” meta-principle could also be applied to laboratory result alerting systems. Nonetheless, the evidence that underpins the principles presented here is valid for medication alerting systems only.

In addition to the results, the method used to build this set of evidence-based usability design principles could also be applied to evidence-based usability design principles for other kinds of technology. However, this method is very time-consuming, and requires in-depth knowledge of the usability of the technology in question if the data are to be analyzed correctly.

### Turning the results into a usable, practical tool for designers and evaluators

The present set of evidence-based usability design principles for medication alerting systems must be made accessible to and usable by designers and evaluators. At present, the principles are presented as a printable table (Table 2) that might not be ideal for optimal use. We intend to use the table to develop tools that present the evidence-based knowledge in a way that suits the needs of the various system designers and evaluators (usability experts, computer scientists, etc.) in various contexts of use (design, evaluation, procurement processes, etc.). With that aim in mind, we have started to identify the needs of medication alerting system designers and evaluators [48]. Accordingly, we developed (i) a checklist that measure the appropriate use of evidence-based principles in the design of medication alerting systems, and (ii) a set of interactive design instructions illustrated by visual representations of good and bad usability practices, in order to help designers make informed design decisions.

This list of usability design principles should help designers to make evidence-based usability design decisions. Nonetheless, and even though we believe that the list is helpful, it is not intended to be used as a stand-alone system or to replace the requirement for expertise in usability and design. Firstly, the present list does not include general design principles for unspecified interactive systems; it must therefore be used in combination with sets of general usability design principles for interactive systems (e.g. [43, 44]). Secondly, several principles require insights into the users’ cognitive tasks and their decision-making processes in order to adjust (for instance) an alert’s format and the moment at which it appears (e.g. #24). Hence, work system and cognitive work analyses [49] must be performed so the principles are applied in an optimal way. Thirdly, principles moderate each other; they must not be applied alone or in an unquestioning manner. Human factors specialists and designers must use their expertise to determine which principles must be applied and how they must be applied, given the characteristics of the alerting system and the setting in which it is implemented in (hospital vs. primary care, for example). In summary, this structured list of usability design principles must be used as a support for expertise and not as a substitute for it.

Applying some of the principles listed here may present specific technical and organizational challenges when seeking to tailor alerts. For instance, the “prioritize the alerts according to patient’s clinical context and the severity of the unsafe event” (#8) principle requires access to valid data on the patient’s clinical context, stay, and treatment. However, these data are often not standardized or structured enough to be used in the alerting system’s set of rules [50]. Further research is needed to overcome these challenges.

Ultimately, presenting designers and evaluators with evidence-based knowledge may help to decrease the occurrence of unforeseen and potentially harmful usability-induced use errors. Nonetheless, one must be aware that improving the usability of an existing system or ensuring that the usability of a system under development is optimal is no guarantee of success. Other issues arising during the development of a medication alerting system (e.g. an error-ridden knowledge base, a poor implementation process, unsuitable settings, etc.) can ruin even optimal levels of usability. Even though it is necessary to consider usability during the design, development, and evaluation of medication alerting systems, one must never neglect the relevant technical, social, and managerial factors that also contribute to the system’s success or failure.

## Conclusions

In the present study, we developed an evidence-based, structured, specific, comprehensive list of usability design principles for medication alerting systems, and then illustrated them with the corresponding usability flaws. This list should help designers and usability experts to gain a better understanding of usability design principles. We expect that the list can be used during the design and evaluation processes of medication alerting systems, in order to prevent usability issues that could have a counterproductive impact on clinicians (e.g. alert fatigue) and potentially harmful outcomes for patients (e.g. errors in medication dosing). Although operational barriers may complicate the deployment and maintenance of the evidence-based usability design principles presented in the present study, our results show that the approach is feasible. Indeed, our approach could be transferred to other health information technologies for the generation of specific lists of evidence-based usability design principles. In this way, designers and evaluators could be provided with tools to help them avoid usability design issues in health information technology and thus decrease the likelihood of unforeseen and potentially harmful usability-induced use errors.

## Additional files

Additional file 1: **Appendix 1.** List of usability design principles identified in the 9 papers and the corresponding usability design principles summarized (for definitions, please refer to Table 2). (DOCX 248 kb)

Additional file 2: **Appendix 2.** The 9 papers’ contributions to the summarized principles. Crosses show that a given principle is mentioned in a paper. The right-hand-most column gives the number of papers mentioning a given principle. The bottom two rows present the number of principles mentioned by each paper and the proportion of the full list of principles mentioned by each paper. It should be noted that the percentages are based on the 58 principles summarized in step 1. Principles #46 and #57 were created after the matching process and therefore were not included here. (DOCX 56 kb)

Additional file 3 **Appendix 3.** Results of the matching between instances of usability flaws (from Marcilly et al. [17]) and the usability design principles summarized in the present study. (DOCX 82 kb)

## Abbreviations

ATC: Anatomical Therapeutic Chemical
CPOE: Computerized Physician Order Entry
EHR: Electronic Health Record
