# Supplementary material for: Evidence-based usability design principles for medication alerting systems
Source: BMC Med Inform Decis Mak. 2018 Jul 24;18:69. doi: 10.1186/s12911-018-0615-9 (PMC6057098; doi:10.1186/s12911-018-0615-9)
Supplement: Supplementary file 1 — Appendix 1. List of usability design principles identified in the 9 papers and the corresponding usability design principles summarized (for definitions, please refer to Table 2). (DOCX 248 kb) [file 12911_2018_615_MOESM1_ESM.docx]

**Additional file 1: Appendix 1.** List of usability design principles identified in the 9 papers and the corresponding usability design principles summarized (for definitions, please refer to Table 2).

| **Reference** | **Excerpt from the papers representing usability design principles** | **Corresponding synthesized usability design principles** | | | | | |
| --- | --- | --- | --- | --- | --- | --- | --- |
| [1] | Managing the specificity, sensitivity and other performance characteristics of alerts is necessary | #1 |  |  |  |  |  |
| [1] | Filtering alerts by increasing the specificity of trigger rules may help to decrease the number of interruptive messages with little evidentiary basis or clinical relevance or those that are redundant | #1 |  |  |  |  |  |
| [23] | reduce the number of disruptive alerts of low clinical value. | #1 |  |  |  |  |  |
| [23] | Suppressing alerts with little evidentiary basis or clinical relevance, or those that are redundant further increases their specificity. | #1 |  |  |  |  |  |
| [19] | avoid over-alerting by improving specificity of alerts and by improving allergy data quality. | #1 |  |  |  |  |  |
| [19] | Drug-allergy checking should include evidence-based cross-sensitivity checking (clearly indicating, and possibly not alerting, if reported cross-sensitivities occur rarely). | #1 |  |  |  |  |  |
| [20] | minimize the overall number of alerts in the system and the frequency with which they activate. | #1 |  |  |  |  |  |
| [4] | increased sensitivity to the needs of the current clinical scenario | #1 |  |  |  |  |  |
| [4] | Combine recommendations for patients with co-morbidities | #10 |  |  |  |  |  |
| [4] | create mechanisms to identify and eliminate redundant, contraindicated, potentially discordant, or mutually exclusive guideline based recommendations for patients presenting with co-morbid conditions or multiple medications. | #10 |  |  |  |  |  |
| [23] | These messages [low severity messages] can also be aggregated and shown together in a single display to be reviewed all at once at a convenient point in the workflow such as at the end, during order signing. The messages can also be sorted and prioritized. | #11 |  |  |  |  |  |
| [20] | alerts should be grouped meaningfully | #11 |  |  |  |  |  |
| [19] | Organizations should customize duplicate checking to decrease the number of clinically insignificant alerts | #13 |  |  |  |  |  |
| [19] | Customers may also want to create additional alerts when appropriate | #13 |  |  |  |  |  |
| [19] | Screen recommendations with expert local clinicians first. | #13 |  |  |  |  |  |
| [19] | Vendors should implement knowledge management tools for their customers’ use. Healthcare organizations that purchase CPOE products should be able to review embedded drug information resources to determine where errors are present, and to decide which specific alerting rules they want to implement. | #13 | #12 |  |  |  |  |
| [19] | Application vendors’ tools should allow provider organizations to make local customizations to the knowledge base, and these customizations should survive vendor product upgrades. | #13 | #12 |  |  |  |  |
| [19] | implement knowledge management tools so that provider organizations can customize purchased drug information, and so customizations persist across version upgrades. | #13 | #12 |  |  |  |  |
| [24] | We are not aware of evidence that demonstrates that it is safe to eliminate DDI alerts for specialists. However, establishing more selective institutional DDI alerting practices overall may relieve much of the alert burden. EHR system architecture should allow institutions to easily make these changes based on clinician characteristics. | #13 | #12 |  |  |  |  |
| [23] | Specificity or sensitivity will likely be improved as the result of consensus meetings between physicians and pharmacists | #13 | #14 |  |  |  |  |
| [1] | Allow access to logs, analyze periodically to increase specificity, sensitivity of alert rules | #14 |  |  |  |  |  |
| [23] | suppressing a drug–drug interaction alert after it has been overridden only once per patient, for example, was not favored by prescribers | #14 |  |  |  |  |  |
| [19] | Pharmacists should personally review on a frequent periodic basis the ignored formulary alerts | #14 |  |  |  |  |  |
| [24] | We do not recommend that institutions eliminate or completely turn off clinically relevant alerts; instead, alerts could be diverted from interruptive to non interruptive form with on-demand access. | #14 |  |  |  |  |  |
| [24] | alerts that do not provide value (measured or perceived) should be suppressed with precision (i.e., increase specificity) without jeopardizing sensitivity. | #14 |  |  |  |  |  |
| [2] | essential to track impact early when supplying decision support by assessing how often suggestions are followed, and then make appropriate midcourse corrections. | #14 | #12 |  |  |  |  |
| [2] | track the frequency of alerts and reminders and user responses and have someone, usually in information systems, evaluate the resulting reports on a regular basis. | #14 | #12 |  |  |  |  |
| [1] | Curate, revise trigger rules ; Periodic reviews of frequently overridden alerts by a committee that includes pharmacists | #14 | #12 |  |  |  |  |
| [1] | Automatic monitoring of overrides should notify administrators when a preset threshold for the number of alerts that are not accepted is reached in any given time period | #14 | #12 |  |  |  |  |
| [1] | System performance should be periodically reviewed by analyzing the logs. | #14 | #12 |  |  |  |  |
| [1] | Reasons for override may also be prompted routinely so that knowledge engineers trying to determine why some alerts are consistently ignored can review override reasons, and analyze them in conjunction with activity logs. | #14 | #12 |  |  |  |  |
| [1] | customization or periodic reviews are necessary. A committee of physicians that includes domain experts and pharmacists for drug-related alerts should periodically revise rules with a focus on frequently overridden alerts and suggest safe and effective ways for either suppressing alerts of low value or changing their presentation format | #14 | #12 |  |  |  |  |
| [23] | Periodic reports on the number of alerts, proportion of overrides and the frequency of pharmacist intervention are necessary for refining trigger rules and making them more specific | #14 | #12 |  |  |  |  |
| [23] | A committee of physicians should periodically revise the rules and suggest safe and effective ways for filtering or changing the presentation format of frequently overridden alerts. | #14 | #12 |  |  |  |  |
| [19] | Analyses of override reasons should occur as part of system quality improvement efforts, and contribute to further reduction of non-essential alerts. | #14 | #12 |  |  |  |  |
| [1] | A periodic review of underutilized or ignored advisory interventions may point to gaps in rules logic or to inadequately updated patient data that trigger irrelevant alerts | #14 | #2 | #12 |  |  |  |
| [1] | Periodically analyze overused, underused orders, consistent alert overrides, integrity of entered data | #14 | #2 | #12 |  |  |  |
| [1] | frequent and consistent overrides may point to outdated or incorrect criteria in decision logic, irrelevancy for particular clinical context (e.g., in case of medication refills or health maintenance reminders for hospitalized patients), erroneous data in the patient record (e.g., medication and allergy lists that are not well maintained) or to inconvenient design of alerts that are either too intrusive or are activated at a point in the clinical workflow when the suggested response cannot be made. | #14 | #2 | #24 | #30 | #6 | #12 |
| [24] | Allow institutional flexibility in determining interruptive vs. non-interruptive DDI alerts | #15 |  |  |  |  |  |
| [24] | As such, we advocate a team approach to managing DDIs | #16 |  |  |  |  |  |
| [21] | Make the System a Team Player | #16 |  |  |  |  |  |
| [24] | DDI decision support applies to all clinicians on the care team: prescriber, pharmacist, nurse, patient, and others | #16 | #17 |  |  |  |  |
| [21] | incorporate functions to support the team awareness about the alert management and its evolution over time (e.g. visible access to how the alert was handled and to the reasons for alert override or rule deactivation if any has been documented) | #17 | #19 |  |  |  |  |
| [21] | provide an indication for all the professionals of the availability of an information | #18 |  |  |  |  |  |
| [23] | The recorded justifications and possibly other annotations also allow nurses and pharmacists to understand the rationale for each override | #19 |  |  |  |  |  |
| [19] | The override reason should allow nurses and pharmacists to understand the rationale for the override. | #19 |  |  |  |  |  |
| [24] | Actions and feedback should be stored for quality improvement efforts and made available for other clinicians to view, such as when this alert is displayed again for the same patient | #19 | #14 |  |  |  |  |
| [1] | Missing, outdated, erroneous or contradictory data must not result in incorrect advice or lower safety | #2 |  |  |  |  |  |
| [1] | when clinical data are aggregated from multiple sources they may need to be ‘‘normalized’’ into a common representational format (common or converted units of measurement, reference range, etc.) and analogous data reconciled by identifying their ‘‘source of truth’’ | #2 |  |  |  |  |  |
| [1] | Shared lists of allergies, medications and problems for each patient should serve as singular reliable sources on which the decision support rules operate across the network | #2 |  |  |  |  |  |
| [1] | Institutions with several EHRs need to integrate multiple instances of allergy lists stored on different networked systems that may contain conflicting or unreconciled information | #2 |  |  |  |  |  |
| [1] | [intermediate variables] may be monitored and automatically updated over time to reflect changes in laboratory results, medications, problems, procedures, passages of time and other data. | #2 |  |  |  |  |  |
| [23] | inappropriate alerts when using data in the EHR that are outdated, not reconciled or inaccurate. | #2 |  |  |  |  |  |
| [19] | Effective drug–drug interaction checks require accurate information about which drugs a patient is taking. | #2 |  |  |  |  |  |
| [1] | Timely maintenance of patient allergy lists and trigger rules can reduce the number of ‘‘false-positive’’ alerts that have low clinical value over time. | #2 | #3 |  |  |  |  |
| [1] | high specificity of alerts is dependent on the quality of the knowledge base for triggering rules and the completeness and accuracy of electronic health records on which the rules operate. | #2 | #3 |  |  |  |  |
| [1] | This data, however, may be missing, incorrect, imprecise, dated or otherwise unreliable. The inference engine (i.e., the set of rules and algorithms that generate advice) needs to function safely in such conditions and prevent the system from giving inappropriate advice | #2 | #4 |  |  |  |  |
| [1] | Another way of reducing the total number of messages presented to a single physician is redirecting them instead to pharmacists, nurses or other staff when appropriate and desirable | #20 |  |  |  |  |  |
| [1] | Messages that prompt for routine actions (e.g., periodic lab tests for patients with chronic conditions) should be offloaded from physician workflows entirely and redirected to support staff. | #20 |  |  |  |  |  |
| [24] | For nonprescribing clinicians, DDI alerts may be deployed as a second check to help ensure that patients receiving interacting drug pairs are being monitored or assessed. | #20 | #18 |  |  |  |  |
| [23] | domain specialists may not need the same level of support as generalists | #21 |  |  |  |  |  |
| [24] | We recommend that general alert content be consistent among various types of clinicians | #21 |  |  |  |  |  |
| [24] | What may differ, however, is how the information is presented to various professionals. The message may be changed based on the context or functions, recognizing that professionals in different settings have different roles, responsibilities, and privileges. | #21 |  |  |  |  |  |
| [21] | have the same display of basic CDS information for the case at hand for all professionals | #21 |  |  |  |  |  |
| [21] | give access upon request to extended information (justification of the rule, attached scientific documentation, etc.) that should be structured depending on the user profile. | #21 |  |  |  |  |  |
| [1] | Pharmacy systems receiving electronic prescriptions should also have their own automatic drug–drug and drug–allergy checking in addition to decision support built into the ordering system | #21 | #20 |  |  |  |  |
| [1] | The networked systems, however, should share the same clinical context so that pharmacists can better evaluate the appropriateness of each prescription. A summary screen containing key patient information, for example, may include flags for all alerts that physicians have overridden, including the rationale and relevant details (e.g., the patient is taking high-risk medications such as warfarin or monoamine oxidase inhibitors) | #22 | #19 |  |  |  |  |
| [2] | Fit into the User’s Workflow. | #23 |  |  |  |  |  |
| [2] | Understanding clinician workflow, particularly when designing applications for the outpatient setting, is critical. | #23 |  |  |  |  |  |
| [2] | Optimal clinical decision support systems should also have the capability to anticipate the subtle ‘‘latent needs’’ of clinicians in addition to more obvious needs. | #23 |  |  |  |  |  |
| [1] | Workflow integration Appropriate sequence of screens, context, type and timing of advice by clinical task | #23 |  |  |  |  |  |
| [20] | it is essential that alerting systems adequately support users’ mental models and correct them as appropriate. | #23 |  |  |  |  |  |
| [20] | The presentation of alert information, and information more generally, should as far as possible match the mental models of the user | #23 |  |  |  |  |  |
| [4] | supports and does not interrupt the clinical workflow | #23 |  |  |  |  |  |
| [2] | Anticipate Needs and Deliver in Real Time. | #23 | #24 |  |  |  |  |
| [2] | bring information to clinicians at the time they need it | #24 |  |  |  |  |  |
| [1] | Tight integration with clinical workflows and presentation of relevant advice at the time and place of decision making | #24 |  |  |  |  |  |
| [19] | different alerts may be presented in different ways, for example, some may be presented at the time of ordering, others at the time of logging on, and others perhaps as automated e-mail messages. | #24 |  |  |  |  |  |
| [24] | We recommend that DDI alert information be displayed at the point of decision making | #24 |  |  |  |  |  |
| [24] | Decision support, including DDI CDS, should be displayed concurrently with the decision process | #24 |  |  |  |  |  |
| [2] | Speed is everything | #25 |  |  |  |  |  |
| [2] | Our goal is sub second ‘‘screen flips’’ | #25 |  |  |  |  |  |
| [1] | they need to rapidly receive advice | #25 |  |  |  |  |  |
| [1] | the acceptable screen transition time is well under a second | #25 |  |  |  |  |  |
| [24] | Present alerts with the need for minimal or no scrolling | #25 |  |  |  |  |  |
| [23] | an alert may be superimposed over an ordering page that contains the list of currently active medications that clinicians may want to see. Dialogs should therefore be movable and resizable. | #26 |  |  |  |  |  |
| [23] | [dialog size] Variable to accommodate content without appearing oversized or dense; collapsing and moving is allowed to see information underneath | #26 |  |  |  |  |  |
| [20] | located in close proximity to the controls and displays relevant to the situation being indicated. | #26 |  |  |  |  |  |
| [23] | The dialog boxes should be displayed over the screen with currently entered orders (presumably the starting point for creating new orders that immediately preceded the alert) to provide sufficient context for the decision and allow clinicians to clearly see the outcome of their actions. | #26 | #41 |  |  |  |  |
| [1] | anticipating follow-up steps in common workflows by providing shortcuts | #27 |  |  |  |  |  |
| [19] | and then return clinicians to their previously intended workflows | #27 |  |  |  |  |  |
| [24] | Alert resolution should be facilitated via on-screen operations | #27 |  |  |  |  |  |
| [24] | There should be as few steps (e.g., keystrokes, mouse clicks, scrolling, window changes) as possible to resolve the potential alert. | #27 |  |  |  |  |  |
| [20] | assess what corrective actions are going to be required by an alert and how the alert is acknowledged and canceled. | #27 |  |  |  |  |  |
| [20] | alerts should cancel and reset in response to the appropriate corrective action rather than requiring an acknowledgment from the operator followed by the corrective action. | #27 |  |  |  |  |  |
| [20] | Alerts which require acknowledgement before the user moves on should be kept to a minimum. | #27 |  |  |  |  |  |
| [22] | streamline the user’s workflow in responding to alerts. | #27 |  |  |  |  |  |
| [22] | minimize the number of steps the user must take in dealing with an alert, as well as to efficiently capture the user’s response to the alert, or intended action. | #27 |  |  |  |  |  |
| [22] | efficient means for carrying out any of the steps described for dealing with an alert | #27 |  |  |  |  |  |
| [22] | use of a simplistic corrective action | #27 |  |  |  |  |  |
| [22] | Through one click located within the alert, the user is able to simultaneously acknowledge having seen an alert, as well as convey an acceptance or rejection of the alert with respect to their patient. | #27 |  |  |  |  |  |
| [19] | The way alerts are presented to providers should be improved in part through differential display based on the severity of the anticipated event. | #28 |  |  |  |  |  |
| [24] | Alerts for the most serious (e.g., contraindicated) DDIs should be easily distinguished from those that are less serious. | #28 |  |  |  |  |  |
| [20] | Alerts of the same level of severity should be perceived as equally urgent, but different from those of a lower severity or purpose. | #28 |  |  |  |  |  |
| [23] | Alerts with lower urgency should be clearly noticeable, placed near the order for which they were triggered | #29 |  |  |  |  |  |
| [23] | Messages about possible interactions that are considered merely informational (i.e., with the lowest severity rating) can be placed in regions on the screen that are not in the focused visual field of the clinician at the moment the order is entered. | #29 |  |  |  |  |  |
| [23] | They [messages merely informational] can be in areas dedicated to warnings, in sidebars or in the main body section and expanded on demand | #29 |  |  |  |  |  |
| [20] | visual alerts must be placed within an operator’s visual field. | #29 |  |  |  |  |  |
| [20] | Visual alerts should be located in the visual field in order of importance, so that the highest priority alerts are located in the stationary field, with lower priority alerts in the eye field and head field. | #29 |  |  |  |  |  |
| [20] | A distinction should be made between alerts related to medications versus those that relate to system errors. | #29 |  |  |  |  |  |
| [24] | We recommend consistent use of color and symbols to aid alert recognition | #29 | #34 |  |  |  |  |
| [24] | Use color as a redundant cue to consistently distinguish more serious from less serious DDIs | #29 | #34 |  |  |  |  |
| [24] | Use symbols consistently to identify more serious from less serious DDIs | #29 | #34 |  |  |  |  |
| [24] | Use color and symbol pairs within and across EHR systems to identify more serious from less serious DDIs | #29 | #34 |  |  |  |  |
| [2] | keep up with the pace of change of medical knowledge | #3 |  |  |  |  |  |
| [19] | knowledge bases upon which monitoring recommendations are made should be evidence-based, with documentation of benefits. | #3 |  |  |  |  |  |
| [19] | adequate knowledge base of drug–disease contraindications. | #3 |  |  |  |  |  |
| [1] | Tiered severity level, interruptive alerts should be reserved only for high severity warnings (of 2–3 levels) | #30 |  |  |  |  |  |
| [1] | Alerts can also be tiered into two or three severity levels and presented in more and less intrusive forms according to importance. | #30 |  |  |  |  |  |
| [1] | [interruption] should be reserved only for high severity warnings and used judiciously | #30 |  |  |  |  |  |
| [1] | [lower importance messages] can be text messages in sidebars that can be read without explicit acknowledgment or for the moment ignored | #30 |  |  |  |  |  |
| [23] | Filtering of alerts means that rules triggering specific intervention modes (e.g., interruptive dialogs or non-intrusive messages) are modified not to activate when certain conditions apply. | #30 |  |  |  |  |  |
| [23] | degree of alert intrusiveness can be adjusted according to their level of importance, allowing only the most severe warnings to interrupt work | #30 |  |  |  |  |  |
| [23] | The most serious warnings still need an explicit response by a clinician but less important alerts are displayed less intrusively on the screen as messages not requiring any actions. | #30 |  |  |  |  |  |
| [23] | Warnings about the most serious interactions are intentionally interruptive | #30 |  |  |  |  |  |
| [19] | Different alerts should be presented depending on whether the patient has mild renal insufficiency, untreated uremia, or is actively undergoing dialysis. Creative approaches to communicating alerts may help, for example, by placing formation about renal and hepatic function “non-interruptively” on usual data entry screens rather than displaying them in separate “pop-up” screens. | #30 |  |  |  |  |  |
| [19] | Degree of severity should influence interruptive versus non-interruptive notification methods. | #30 |  |  |  |  |  |
| [24] | We recommend reserving interruptive alerts (i.e., those requiring action by clinicians before proceeding) for the most serious DDIs. | #30 |  |  |  |  |  |
| [24] | Generally, if a less likely or less serious DDI is detected, the healthcare professional should be able to receive the relevant information through non-interruptive CDS such as a link or Infobutton or retrospective surveillance reports intended for ongoing patient monitoring. During surveillance, if the presence of a DDI clinical consequence is detected, an interruptive alert or report may be presented to the healthcare professional responsible for that patient’s ongoing care. | #30 |  |  |  |  |  |
| [24] | Use non-interruptive alerts for less likely or less serious DDIs to facilitate on-demand workflows | #30 |  |  |  |  |  |
| [20] | only safety-critical events are classified as unsafe and requiring an alert | #30 |  |  |  |  |  |
| [4] | intrusiveness proportional to the importance of the information | #30 |  |  |  |  |  |
| [23] | committee consensus on how many severity levels will be used, which alerts are designed as interruptive dialogs and which rules may be suppressed by filtering. | #30 | #15 |  |  |  |  |
| [1] | Unobtrusive reminders may be designed as flags in names lists; prioritized and color-coded messages in reserved screen areas | #30 | #29 |  |  |  |  |
| [23] | Systematic and consistently applied nomenclature and display formalisms | #31 |  |  |  |  |  |
| [24] | We recommend greater uniformity and consistency in DDI alert presentation across systems | #31 |  |  |  |  |  |
| [24] | we recommend alert presentation consistency within and across different EHR systems | #31 |  |  |  |  |  |
| [1] | Messages within alerts should be generally shorter than ten words | #32 |  |  |  |  |  |
| [1] | Concise language Place important words first, details later in the sentence. Display ten words or less and provide a link to the full text | #32 |  |  |  |  |  |
| [1] | Content should be limited to 1–2 lines, with a justification separated by white space | #32 |  |  |  |  |  |
| [1] | Multi-word button labels and verbose messages make the selection of the intended response by perceptual judgment difficult and generally do not add clarity. | #32 |  |  |  |  |  |
| [23] | Concise and clear recommendations are the most effective | #32 |  |  |  |  |  |
| [23] | Concise and unambiguous statements and directions. Directly visible messages are shorter than 10 words; details available on demand (links). | #32 |  |  |  |  |  |
| [23] | concise wording and justification | #32 |  |  |  |  |  |
| [23] | [have a succinct explanation of the interaction] (…) and generally shorter than ten words. | #32 |  |  |  |  |  |
| [19] | clearly written “to-the-point” guidelines with links to additional information if desired | #32 |  |  |  |  |  |
| [19] | The CDS UI should present information clearly and concisely | #32 |  |  |  |  |  |
| [19] | create concise and actionable alert messages | #32 |  |  |  |  |  |
| [24] | Information should be presented in concise language, with minimal text. Also, by minimizing the amount of text initially presented | #32 |  |  |  |  |  |
| [24] | Use concise language and minimal text | #32 |  |  |  |  |  |
| [1] | Brief and clear recommendations are the most effective. Language used in messages needs to be succinct and use instantly recognizable terms that can be accurately interpreted in a single reading | #32 | #35 |  |  |  |  |
| [1] | The most salient part of a message (e.g., dose, patient weight) should be shown before any other supporting information is given. | #33 |  |  |  |  |  |
| [24] | the most critical information should be presented on the top-level screen of the alert, with linked information accessible on-demand about background and secondary considerations. | #33 |  |  |  |  |  |
| [24] | Citations to the primary literature are not essential in the dialog box (top-level screen) but should be available on-demand for the clinician to “drill down” and access this content via embedded linked information or relevant websites (e.g. PMID for PubMed). | #33 |  |  |  |  |  |
| [20] | Signal words are the header terms often found at the top of warning labels, terms such as ‘danger’, ‘warning’, and ‘caution’. | #33 |  |  |  |  |  |
| [20] | instruction and hazard statements are the most important to include. | #33 |  |  |  |  |  |
| [23] | The two drug names are the most important information on the screen; they are therefore the most prominent screen artifacts to draw visual attention. | #33 | #37 |  |  |  |  |
| [23] | It [severity level] needs to be the most visually prominent item on the screen and clearly communicated by dedicated “code” words reserved for each level (…) and used consistently for all warnings in the entire system. | #33 | #39 | #34 | #29 |  |  |
| [1] | Tests, procedures, orders and sets, alerts, menus should use consistent language | #34 |  |  |  |  |  |
| [1] | All terms, including the names of laboratory tests, procedures and order sets, need to be used consistently across menus, lookup tables and in advisory messages generated by decision support interventions | #34 |  |  |  |  |  |
| [1] | A nomenclature of conceptual categories should also be consistent and unambiguous. For example, if alerts are classified into three severity tiers such as ‘‘critical,’’ ‘‘significant’’ and ‘‘caution,’’ the same terms need to be used in all alerts, messages and textual references | #34 |  |  |  |  |  |
| [1] | Similarly, consistent color coding, use of highlights and fonts and visual hierarchies needs to be maintained within all modules of a system and preferably across all interoperable systems | #34 |  |  |  |  |  |
| [1] | Prompts and instructions should employ a consistent wording style. | #34 |  |  |  |  |  |
| [1] | well-defined and consistently applied terminology for observations, assessments and medical concepts. Terms for adverse reactions, problems, procedures and other activities or items as well as conceptual categories describing groups of related data should be standardized across all networked systems | #34 |  |  |  |  |  |
| [1] | Consistent terminology [for] adverse reactions, problems, procedures, medical concepts, assessments, drug and drug class names | #34 |  |  |  |  |  |
| [1] | Meaningful color sets matched across all systems | #34 |  |  |  |  |  |
| [24] | We recommend the use of consistent terms and definitions to indicate the potential seriousness of the DDI. | #34 |  |  |  |  |  |
| [24] | We recommend the use of consistently phrased alerts and terms throughout and across DDI decision support systems. | #34 |  |  |  |  |  |
| [24] | Use consistent terms and definitions for DDI classification to indicate potential seriousness in a dialog box. | #34 |  |  |  |  |  |
| [24] | Use consistent phrasing and terms throughout and across all DDI CDS systems | #34 |  |  |  |  |  |
| [24] | Meanings of color and symbols should be clear to all clinicians. | #34 | #21 |  |  |  |  |
| [23] | Terminology should follow local conventions but needs to be applied consistently. | #34 | #35 |  |  |  |  |
| [23] | “Discontinue” or “D/C,” should not be used to cancel an order that has not yet been completely entered—the one that triggered the alert. The term applies to the creation of a new order to stop an active medication and may be therefore confusing. | #34 | #35 |  |  |  |  |
| [23] | [have a succinct explanation of the interaction] (…) using recognizable and accepted terms, must be easily interpretable | #35 |  |  |  |  |  |
| [23] | Terminology should follow established local conventions but labels with more than two words (e.g., “continue with current order” or “override alert and continue order”) are excessively long and may not clearly convey the effect of the action. | #35 | #32 |  |  |  |  |
| [24] | We recommend that seven components be integrated into alerting systems for DDI warnings: (1) drugs involved; (2) seriousness; (3) clinical consequences; (4) mechanism of the interaction; (5) contextual information/modifying factors; (6) recommended action(s); and (7) evidence. | #36 |  |  |  |  |  |
| [24] | Prototypes should include all seven alert components we recommend with the objective of unambiguous and intuitive design | #36 |  |  |  |  |  |
| [24] | 1. Drugs involved 2. Clinical consequence (and frequency) 3. Seriousness 4. Recommended action 5. Contextual information/Modifying factors 6. Mechanism of interaction 7. Evidence | #36 |  |  |  |  |  |
| [24] | To clearly identify interacting drug pairs, we recommend using the medication name as ordered as well as generic ingredient names, which is especially important or combination products. | #37 |  |  |  |  |  |
| [24] | Interacting drugs should be clearly identified in a dialog box | #37 |  |  |  |  |  |
| [24] | Inappropriate listing of DDIs by pharmacologic or therapeutic class can lead to the clinician incorrectly assuming that the DDI applies to the entire class of medications. This does not preclude mentioning pharmacologic or therapeutic “class-based” effects, when appropriate (e.g., as a recommended action, avoid the entire pharmacologic class). | #37 |  |  |  |  |  |
| [24] | Use the medication name as ordered as well as generic ingredient drug names when identifying the interaction | #37 |  |  |  |  |  |
| [24] | Critical information should be present within an alert, including names of the interacting drugs. Specifying interacting agents should make it immediately clear for what the DDI alert is being displayed. | #37 | #33 |  |  |  |  |
| [19] | Alerts should present the names of the interacting drugs, a brief (one-line) description of the interaction, optional links to more detailed information, and a menu for potentially appropriate actions in response to the alert. | #37 | #38 | #32 | #49 | #42 | #33 |
| [20] | a warning label should, if possible, have four information components: a signal word to indicate the priority of the alerts (ie, ‘note’, ‘warning’, or ‘danger’), a statement of the nature of the hazard, an instruction statement (telling the user how to avoid the danger), and a consequence statement (telling the user what might happen if the instruction information is ignored). | #37 | #38 | #39 | #42 |  |  |
| [24] | Clearly conveying the reason for the alert and why it was assigned a given level of seriousness is useful for the clinician | #37 | #39 |  |  |  |  |
| [23] | Identification of an alert as a “drug–drug interaction”, for example, may be added to the top banner of the dialog box but the most important attribute is the severity level. | #37 | #39 | #33 |  |  |  |
| [23] | Rule that triggered the alert and medical consequence are briefly described and a link to detailed explanation (monograph) is attached. | #37 | #43 | #38 | #40 |  |  |
| [23] | (have a succinct explanation of the interaction) and its consequence, | #38 |  |  |  |  |  |
| [24] | The potential adverse clinical outcome(s) for the patient taking interacting drugs should be clearly described | #38 |  |  |  |  |  |
| [24] | Clearly describe the potential clinical consequences of co-prescribing the interacting drugs in a dialog box | #38 |  |  |  |  |  |
| [24] | Alerts should describe the clinical consequences (e.g. hyperkalemia, QT prolongation, reduced efficacy leading to transplant rejection), rather than couch them in generic terms of a ‘safety risk ’ or warning of unspecified dangers | #38 |  |  |  |  |  |
| [24] | When available, present the frequency or incidence of the clinical consequence associated with co-prescribing the interacting drugs | #38 |  |  |  |  |  |
| [20] | Visual alerts can contain textual information with specific details about the unsafe event. | #38 |  |  |  |  |  |
| [24] | When available, the frequency or incidence of adverse drug events (ADEs) associated with a specific DDI, as well as predisposing risk factors for the adverse outcomes, may be useful for estimating the risk for an individual patient. | #38 | #41 |  |  |  |  |
| [20] | For visual warnings, priority is normally indicated by color. | #39 | #29 |  |  |  |  |
| [20] | In addition to color the use of a signal word is particularly relevant to text-based alerts | #39 | #29 |  |  |  |  |
| [1] | Systems should follow a pattern of ‘‘graceful degradation’’ and continue to function at a reduced level of performance when components fail or data are not available | #4 |  |  |  |  |  |
| [1] | reconcile multiple medication, problem, allergy lists | #4 |  |  |  |  |  |
| [19] | Organizations that cannot provide a single allergy database should link multiple ones. | #4 |  |  |  |  |  |
| [1] | Increase specificity by evaluating more EHR data in trigger rules and suppress ‘‘false positives’’ | #4 | #1 |  |  |  |  |
| [1] | Increasing the variety of information sources that are available to the decision rules engines to access and consider—medical and laboratory claims, test results, feedback from physicians, and self-reported data from patients who are enrolled in disease management or complete health risk assessments—is likely to greatly increase the specificity and credibility of clinical alerts in the future and increase the response of clinicians to potentially risky medication | #4 | #6 |  |  |  |  |
| [23] | have a succinct explanation of the interaction | #40 |  |  |  |  |  |
| [24] | The alert should describe the mechanism of the DDI, when known | #40 |  |  |  |  |  |
| [24] | Alert messages should describe the mechanism of the interaction, possibly by accessing embedded links | #40 |  |  |  |  |  |
| [24] | the DDI mechanism and supporting evidence may not be necessary to view on the primary alert interface as long as the information is easily accessible via links | #40 | #43 |  |  |  |  |
| [1] | As the actual indications may differ from those considered by the decision logic, contextual information from the record needs to be evident to support the relevance of the advice. | #41 |  |  |  |  |  |
| [23] | Appropriate contextual information from the patient record should be made available on demand (e.g., via a link) | #41 |  |  |  |  |  |
| [23] | The linked information source may show, for example, the last measured drug serum or creatinine levels, and other interacting drugs that taken together may further support the recommendation | #41 |  |  |  |  |  |
| [23] | Clinical context shows relevant values from the patient record with a link to access further details. | #41 |  |  |  |  |  |
| [24] | include any predisposing factor information (e.g., co-morbidity, lab results) to add relevance and to facilitate alert processing | #41 |  |  |  |  |  |
| [24] | Contextual predisposing factor information (e.g., co-morbidity or lab values) that adds relevance and facilitates response to DDI alerts should be displayed in a dialog box | #41 |  |  |  |  |  |
| [4] | Summarize patient-level information | #41 |  |  |  |  |  |
| [4] | make all key data needed for optimal decision-making available to each decision maker | #41 | #21 | #22 |  |  |  |
| [24] | adjudication of DDI alerts should be based on an assessment of patient-specific factors such as age, predisposing diseases, pharmacogenomic phenotype, and the specific drug regimen(s), such as dose, route, duration of therapy, sequence of initiating co-therapy, and timing of co-administration. This information should be included in the alerting logic or presented within the alert display. | #41 | #6 |  |  |  |  |
| [2] | displaying suggested orders | #42 |  |  |  |  |  |
| [2] | physicians strongly resist suggestions not to carry out an action when we do not offer an alternative | #42 |  |  |  |  |  |
| [1] | Advice rather than command | #42 |  |  |  |  |  |
| [1] | avoid recommendations that are controversial. Rather, advice should be given only for aspects of care in which there is little disagreement on appropriate management | #42 |  |  |  |  |  |
| [1] | Systems should therefore formulate advisory messages in the manner of highlighting potential or actual problems that require attention and suggest therapeutic opportunities rather than imposing strict, inflexible and unsolicited dictates. However, merely giving an assessment without recommending an action and providing a convenient way to either carry out or disregard it is generally not an effective way to change behavior. | #42 |  |  |  |  |  |
| [23] | An alternative drug to the one being ordered may be suggested as a third option. | #42 |  |  |  |  |  |
| [23] | Accurate suggestions of drug alternatives need to include dose and frequency but those may depend on clinical context. However, both medications must be considered | #42 |  |  |  |  |  |
| [23] | Ancillary order (ECG 12-lead) is included but not mandatory. | #42 |  |  |  |  |  |
| [23] | Brief instructions (monitor ECG daily) are included. | #42 |  |  |  |  |  |
| [19] | offering a non-controversial suggested alternative within the alert window. | #42 |  |  |  |  |  |
| [19] | include a link to information describing institution-specific guidelines for restricted medications. | #42 |  |  |  |  |  |
| [24] | if monitoring is recommended, then it should be actionable from the DDI alert interface. | #42 |  |  |  |  |  |
| [24] | The alert should provide strategies for mitigating potential harm | #42 |  |  |  |  |  |
| [24] | Recommended actions should be as specific as possible, but account for a wide range of patient factors | #42 |  |  |  |  |  |
| [24] | For many types of DDIs, there may be several actions that are clinically appropriate, and in these cases, CDS systems should present multiple suggestions along with details on when each action might be warranted. | #42 |  |  |  |  |  |
| [21] | Make the System a Clinicians’ Partner | #42 |  |  |  |  |  |
| [22] | An alert using intelligent corrective actions would contain a response such as, ‘Continue with warfarin order AND reduce dose by 33e50%.’ | #42 |  |  |  |  |  |
| [22] | fail-safe mechanism in which the system is capable of monitoring whether or not the user followed through with the intended action, the third item in this principle. If the user did not complete intended actions, the system would notify the user | #42 |  |  |  |  |  |
| [24] | CDS systems should be able to present a list of suggestions that could be locally customizable to take into account formulary and other organizational factors | #42 | #13 |  |  |  |  |
| [24] | Guidance on strategies to mitigate potential harm (e.g., dose modification, order cancellation, ordering an alternate medication, or monitoring/surveillance actions) may be presented in the dialog box (top-top level screen) or be accessible on-demand from the dialog box as linked information. | #42 | #33 |  |  |  |  |
| [1] | Highlight potential problems, safety hazards, suggest actions – not directives | #42 | #37 | #38 |  |  |  |
| [23] | clear response options with controls placed close to relevant text | #42 | #49 | #26 |  |  |  |
| [1] | Alternatives rather than stops Suggest alternatives to audited actions, provide direct links to carry them out | #42 | #50 |  |  |  |  |
| [2] | links to referential information supporting the decision support… comprehensive | #43 |  |  |  |  |  |
| [1] | Cultivation of trust, Avoid black box advice, maintain high specificity, context, justification | #43 |  |  |  |  |  |
| [1] | Systems need to avoid the impression of a ‘‘black box’’ giving advice that cannot be subjectively evaluated. | #43 |  |  |  |  |  |
| [1] | A link to further evidence may also be included as clinicians often contend that more information should be accessible | #43 |  |  |  |  |  |
| [1] | A drug alert suggesting a dosing change, for example, may include a link to additional details on why the advisory was shown, further supporting evidence from academic literature and guidelines | #43 |  |  |  |  |  |
| [1] | (A drug alert suggesting a dosing change, for example, may include a link to) a contact to local authorities responsible for the explanation of CDS rationale. | #43 |  |  |  |  |  |
| [19] | knowledge should be evidence-based supported by references; when this is not possible, evidence should be based on experts’ consensus opinion about accepted best practices, and referenced as such. | #43 |  |  |  |  |  |
| [19] | Links should exist to enable clinicians to review the evidence basis for automated drug information, both as bibliographic references and as text summaries of evidence. | #43 |  |  |  |  |  |
| [24] | but alerts should provide some information related to the strength and source of evidence | #43 |  |  |  |  |  |
| [24] | Information related to the strength (or category) of supporting evidence should be easily accessible on-demand | #43 | #33 |  |  |  |  |
| [24] | It may be possible to communicate evidence through a set of symbols, letters and/or numbers supported through use of a clear grading system | #43 | #34 |  |  |  |  |
| [19] | clinicians should be aware of what is not covered by the alerting system. | #44 |  |  |  |  |  |
| [24] | Estimated frequencies of DDIs are not usually known and likely to be underreported and therefore this field will remain largely unpopulated in the near term for many interactions. If this information is not known, that should be stated. | #44 | #47 |  |  |  |  |
| [1] | An explanation of medical logic, including formulas for calculating values, should be accessible on demand so that the justification for alerting is transparent and verifiable. | #45 |  |  |  |  |  |
| [23] | Triggering medical logic needs to be apparent and outlined in a few words accompanied by a link to further evidence | #45 | #43 |  |  |  |  |
| [20] | Documentation of alarm philosophy should include a catalog of unsafe events, an indication of the level of priority (based on the severity of the consequences) of an alert, a description of the logic underpinning the classification of an event as unsafe, and a description of the specific alerts indicating each unsafe event. | #45 | #47 |  |  |  |  |
| [20] | (Alert philosophy: This should specify (as a minimum)) (…)how many priorities there should be for each category of risk. | #47 |  |  |  |  |  |
| [20] | require an explicit definition of what is meant by a safety-critical event | #47 |  |  |  |  |  |
| [20] | Alert philosophy: This should specify (as a minimum) which categories of problems should be included in the alerting system | #48 |  |  |  |  |  |
| [2] | make it easy for a clinician to ‘‘do the right thing.’’ | #49 |  |  |  |  |  |
| [1] | Messages within alerts should be (…) accompanied by an immediately actionable item | #49 |  |  |  |  |  |
| [23] | The alert box should include an immediately actionable item | #49 |  |  |  |  |  |
| [23] | Controls are in the proximity of corresponding (target) items | #49 |  |  |  |  |  |
| [19] | allow clinicians to act on alerts directly from the alert screen when possible | #49 |  |  |  |  |  |
| [24] | Actionable items should be available within a dialog box to facilitate acceptable DDI alert resolution | #49 |  |  |  |  |  |
| [21] | incorporating functions supporting the dialog between the CDSS and the clinician (e.g. acknowledgment / de-activation of the CDSS alert). | #49 |  |  |  |  |  |
| [4] | make it easier for the clinician to take action on the information provided. | #49 |  |  |  |  |  |
| [22] | provide intelligent corrective actions within the alert. | #49 |  |  |  |  |  |
| [22] | by assisting the user in efficiently accepting or rejecting an alert and carrying out recommended actions. | #49 |  |  |  |  |  |
| [1] | take an appropriate action at a convenient point in the workflow without extraneous effort or delay. | #49 | #27 |  |  |  |  |
| [1] | Buttons (order or cancel) with simple labels, action links to additional options (alternatives) | #49 | #27 |  |  |  |  |
| [24] | Actionable alert items should be intuitive and require as few steps as possible to resolve | #49 | #27 |  |  |  |  |
| [20] | the corrective actions should be easy to perform | #49 | #27 |  |  |  |  |
| [1] | Interruptive dialogs should have simple and clearly defined response options, such as [Order] and [Cancel] buttons and a very concise justification. | #49 | #32 | #52 | #50 |  |  |
| [23] | An action link with the [alternative] drug name may be placed on the dialog box | #49 | #50 |  |  |  |  |
| [24] | important to provide guidance on strategies to mitigate potential harm. There are many possible courses of action, such as dose modification, order cancellation, ordering an alternate medication, or monitoring/surveillance actions | #49 | #50 | #52 | #53 |  |  |
| [24] | clinicians should be able to select from a list of actionable choices. These may include: discontinue one or more active medications, cancel the order, modify the dose, provide patient education, and order labs. | #49 | #51 | #50 | #52 | #54 | #53 |
| [23] | Time intervals between interacting drugs should also be considered as earlier-prescribed drugs may have completely metabolized by the time a contraindicated drug is entered. | #5 |  |  |  |  |  |
| [19] | Improve alerting capabilities to detect when patients are overdue for testing and to notify the patient or clinicians appropriately. | #5 |  |  |  |  |  |
| [21] | adapting its behavior to the evolution of the outcome at risk over time (i.e. take into account the evolution of the targeted lab values to filter the rules and adapt its severity) | #5 |  |  |  |  |  |
| [1] | Complex rules may also be needed to inform clinicians ordering potassium chloride (drip or bolus) when the patient already has another active order for potassium and when there has not been a serum potassium value recorded in the past 12 hours, or the most recent value is greater than 4.0 | #5 | #6 |  |  |  |  |
| [23] | Clicking the link should close the dialog box and open a standard ordering form with the appropriate fields prepopulated with new values | #50 |  |  |  |  |  |
| [23] | Alerts need to clearly state that the existing order will be discontinued if the new one is finalized. | #50 |  |  |  |  |  |
| [19] | display a pop-up alert when the clinician attempts to order a non-formulary drug, while providing a selectable list of alternative formulary medications | #50 |  |  |  |  |  |
| [22] | Selecting this option would simultaneously accept the alert and direct the user back to the medication ordering window where the user can adjust the dose appropriately. | #50 |  |  |  |  |  |
| [23] | The first choice [ordering a new drug] should close the dialog box, create a discontinue order for the existing drug and open a pre-populated entry from for the new one. | #50 | #51 |  |  |  |  |
| [22] | would be capable of automatically discontinuing an existing drug directly from the alert, as well as replacing an order for a different drug within the same class | #50 | #51 |  |  |  |  |
| [23] | binary choice between (a) ordering the new drug while simultaneously discontinuing the existing drug of the interacting pair, and (b) canceling the new order. | #50 | #51 | #52 |  |  |  |
| [24] | This may include discontinuing the preexisting, currently active medication, canceling the order “in process” that is being entered, modifying the order being entered, modifying the pre-existing active medication, adding orders for monitoring, or other actions as justified by the specific DDI alert | #50 | #51 | #52 | #53 |  |  |
| [22] | The actions may be: to continue with the order unadjusted, to continue with the order but adjust the dose of one of the drugs, to continue with the order but switch to a different drug within the same class, to discontinue an existing drug and place the order for the new drug, or to cancel the order for the new drug and keep the existing drug. | #50 | #51 | #52 | #58 |  |  |
| [1] | In some instances, when an alternative medication or test is offered, a link (or a button) may be added that closes the dialog and populates appropriate fields in an open order form with the suggested values. | #50 | #53 |  |  |  |  |
| [23] | The second choice [cancelling the order] should close the dialog box and place the focus back on the drug ordering screen. | #52 |  |  |  |  |  |
| [24] | clinicians could delay the alert for a predetermined amount of time (“snooze” function) or send the alert (defer or forward) to a different clinician. | #55 | #56 |  |  |  |  |
| [23] | override an alert may be asked to give a reason for not following the advice | #58 |  |  |  |  |  |
| [23] | The most common override reasons should be selectable from a list of no more than three or four with a single click | #58 |  |  |  |  |  |
| [23] | Override reason selections can be made mandatory for the most critical alerts but otherwise optional | #58 |  |  |  |  |  |
| [23] | differentiate the level of required effort to override severe interactions by requiring a secondary confirmation action. | #58 |  |  |  |  |  |
| [23] | the hard stop is designed as a persistent checkbox selection to discontinue the existing drug. A less restrictive option may allow deselecting the checkbox but still require a subsequent confirmation to override. | #58 |  |  |  |  |  |
| [23] | Override reasons in a selection lists have 1–2 words; lists should contain less than five items. | #58 |  |  |  |  |  |
| [23] | Override is possible with one extra click (uncheck “Discontinue”) for second tier alerts; not possible (if so designed) for critical alerts. | #58 |  |  |  |  |  |
| [23] | Reason for override is selectable by one click but not mandatory. | #58 |  |  |  |  |  |
| [23] | do not allow the overriding of possibly life threatening interactions and the clinician is required to either cancel the new order or discontinue the pre-existing one (a “hard stop”) | #58 |  |  |  |  |  |
| [19] | provide a coded override reason whenever he or she overrides drug-allergy alert. | #58 |  |  |  |  |  |
| [24] | When a clinician believes an alert is not relevant for a particular patient, there should be an option to reject the information and provide rationale/comments for feedback. | #58 |  |  |  |  |  |
| [24] | Override actions must be codified and explicit | #58 |  |  |  |  |  |
| [23] | The dialog box, at minimum, offers a way to continue ordering (i.e., override the warning) or to cancel the order in progress (i.e., accept the suggestion) by clicking respective buttons. | #58 | #50 | #52 |  |  |  |
| [1] | If a potential interaction did not result in problems for a specific patient in the past, physicians should be able to suppress the alert for subsequent dose adjustments to avoid redundant messages | #59 |  |  |  |  |  |
| [1] | Systems should be able to, where appropriate, suppress alerts at the time of renewal of previously tolerated medication combinations for the same patient. | #59 |  |  |  |  |  |
| [23] | Redundant alerts can be suppressed when dose adjustments are entered for a specific patient and at times when a previously tolerated medication combination for the same patient is renewed. | #59 |  |  |  |  |  |
| [23] | Physicians may be allowed to turn off individual alerts, with caveats, based on their practice, knowledge and comfort level | #59 |  |  |  |  |  |
| [23] | Clinicians may suppress alerts for medications that a patient had previously received and tolerated. | #59 |  |  |  |  |  |
| [1] | use contextual information to refine rules | #6 |  |  |  |  |  |
| [1] | More complex patient states (e.g., ‘‘patient is on anticoagulation therapy’’) can be created with sophisticated data-driven derivations that trigger more extensive and more specific interventions. | #6 |  |  |  |  |  |
| [1] | Drug interaction alerts should be primarily patient-specific by taking into account age, gender, body weight, allergies, mitigating circumstances, drug serum levels, renal function and co-morbidity | #6 |  |  |  |  |  |
| [1] | Combining pharmacology and laboratory data into decision rules provides a powerful tool to guide initial drug choice (i.e., drugs where there are laboratory-based indications and contraindications), drug dosing (renal or hepatic, blood level–guided adjustments), laboratory monitoring (laboratory signals of toxicity, baseline and ongoing monitoring), laboratory result interpretation (drug interfering with test) and for broader quality improvement (surveillance for unrecognized toxicity, monitoring clinician response delays) | #6 |  |  |  |  |  |
| [23] | An effective filtering method is to add to the decision logic, along with general drug–drug interaction rules, additional data from the EHR and thus making the rules more patient-specific. | #6 |  |  |  |  |  |
| [19] | CPOE application must have access to the patient’s previous laboratory results. | #6 |  |  |  |  |  |
| [1] | Alerts should be sensitive to clinical context by incorporating more patient-specific data into trigger rules, provide clear, unambiguous information display and carefully calibrate intrusiveness to be proportional to their level of importance. | #6 | #30 |  |  |  |  |
| [23] | Rules that trigger alerts can also be filtered and prioritized to suppress low-severity warnings by using more sophisticated algorithms that integrate patient context and provider-specific data into the decision logic | #6 | #7 |  |  |  |  |
| [1] | Facilitate manual corrections or additions of data in the EHR as part of response actions to alerts | #60 |  |  |  |  |  |
| [1] | Frequently overridden allergy alerts may include a link to remove the allergy from a list in the patient’s record. | #60 |  |  |  |  |  |
| [1] | Prompt for EHR edits Include a link to edit allergy and medication lists in alerts that are frequently overridden | #60 |  |  |  |  |  |
| [1] | If a record does not accurately represent what the clinician knows to be true about the patient, many alerts would appear irrelevant and will be overridden. Corrections can be facilitated by prompting clinicians to update when a specific alert is being consistently overridden. | #60 |  |  |  |  |  |
| [1] | a link to edit the allergy list should be shown in the alert. When reasons are not required to be entered, an algorithm may decide after repeated overrides to prompt the clinician and provide a link to open the record for editing. | #60 |  |  |  |  |  |
| [1] | Conversely, entering a newly captured allergy should also be prompted if it can be inferred that an allergic reaction may have occurred, | #60 |  |  |  |  |  |
| [1] | Automatic prompts and requests for entering additional data need to be used sparingly, however | #60 |  |  |  |  |  |
| [19] | Removal of an allergy from the allergy list should be facilitated when a physician overrides a drug-allergy alert. | #60 |  |  |  |  |  |
| [1] | Although identical or same-class medications usually should not be prescribed for a patient, rules that check for multiple orders must accommodate cases in which such orders are appropriate while still ensuring safety, such as when more than one analgesic is ordered on an as-needed basis or when a patient may require two different antibiotics or more than one type of anticoagulant. | #7 |  |  |  |  |  |
| [19] | Duplicate alerts should fire selectively. For example, a heparin bolus and a heparin drip entered within the same ordering session are likely intentional duplications. | #7 |  |  |  |  |  |
| [21] | adapting its behavior according to a subset of relevant actions taken by clinicians | #7 |  |  |  |  |  |
| [1] | The usefulness of concurrent alerts needs to be evaluated; those that do not absolutely contribute to improving the prescribing process should be suppressed or deemphasized. Those that remain need to be prioritized by severity so that, for example, a low-importance allergy interaction does not conceal a therapy duplication warning. | #8 |  |  |  |  |  |
| [23] | The relative priority of concurrent alerts needs to be evaluated and those that do not absolutely contribute to improving the prescribing process should be suppressed or shown as low-importance messages | #8 |  |  |  |  |  |
| [23] | For example, a system could automatically prioritize recommendations according to a multi-attribute utility model by combining patient and provider-specific data. Age, gender, body weight, mitigating circumstances, drug serum levels, renal function and co-morbidity may modify the severity of expected interaction for that patient and the system then selects appropriate warning level. | #8 |  |  |  |  |  |
| [19] | alert the clinician only when reasonable dosing parameters have been exceeded | #8 |  |  |  |  |  |
| [20] | Recommendations to curb false alarm rates include moving from ‘boundary based’ alarm strategies (whereby an alarm sounds when a given parameter exceeds pre-set limits) to intelligent alarm monitoring systems that monitor several parameters simultaneously and use fuzzy logic-based algorithms to initiate an alert. | #8 |  |  |  |  |  |
| [20] | When developing an alert philosophy, one must carefully consider the threshold of the alert. | #8 |  |  |  |  |  |
| [20] | A well-documented alarm philosophy is necessary to guide decision-making and ensure consistency in alerting. | #8 |  |  |  |  |  |
| [20] | Visual alerts should be prioritized, and prioritization goes hand in hand with hazard matching as a warning implementation strategy. | #8 |  |  |  |  |  |
| [4] | Prioritize and filter recommendations to the user | #8 |  |  |  |  |  |
| [4] | prioritize recommendations according to a multi-attribute utility model by combining patient- and provider-specific data to take into account expected mortality or morbidity reduction, patient preferences and life style, cost to the individual or organization, effectiveness of the test or therapy, how the patient might tolerate the recommended intervention, location in the clinician’s workflow, insurance coverage, genetic and genomic considerations, clinician’s past performance, and other factors. | #8 |  |  |  |  |  |
| [23] | assign alerts to interaction severity categories, or “tiers,” and to control how they are presented to clinicians. | #8 | #28 |  |  |  |  |
| [19] | differentiate among anticipated severities of conditions so that the most appropriate warning mechanism can be used. | #8 | #28 |  |  |  |  |
| [20] | Low priority alerts: These should be avoided or classified as ‘information only’ indicators. Although from a safety point of view more alerts are seen as safer, in practice the reverse is true. | #8 | #28 |  |  |  |  |
| [20] | Prioritization of alerts: This should probably include three levels: low, medium, and high and should be coded using word, color, shape, position on screen, and other indicators known to influence urgency. | #8 | #29 | #39 |  |  |  |
| [1] | Two or three severity levels are generally sufficient to assign advisories into appropriate categories of visual saliency and intrusiveness and improve the overall rate of compliance They can be designated as ‘‘high,’’ ‘‘moderate’’ and ‘‘low’’ or simply as ‘‘critical’’ and significant’’ and appropriately color-coded. | #8 | #30 | #39 |  |  |  |
| [1] | Rules that trigger specific alerts can be filtered to suppress low-severity drug–drug interactions, or be prioritized and made more specific by combining patient and provider-specific data. | #8 | #6 | #7 |  |  |  |
| [1] | Multiple alerts for a single order should be prioritized, deemphasizing low–severity alerts | #8 | #9 | #11 |  |  |  |
