# Supplementary material for: Evidence-based usability design principles for medication alerting systems
Source: BMC Med Inform Decis Mak. 2018 Jul 24;18:69. doi: 10.1186/s12911-018-0615-9 (PMC6057098; doi:10.1186/s12911-018-0615-9)
Supplement: Supplementary file 2 — Appendix 2. The 9 papers’ contributions to the summarized principles. Crosses show that a given principle is mentioned in a paper. The right-hand-most column gives the number of papers mentioning a given principle. The bottom two rows present the number of principles mentioned by each paper and the proportion of the full list of principles mentioned by each paper. It should be noted that the percentages are based on the 58 principles summarized in step 1. Principles #46 and #57 were created after the matching process and therefore were not included here. (DOCX 56 kb) [file 12911_2018_615_MOESM2_ESM.docx]

**Additional file 2: Appendix 2.** The 9 papers' contributions to the summarized principles. Crosses show that a given principle is mentioned in a paper. The right-hand-most column gives the number of papers mentioning a given principle. The bottom two rows present the number of principles mentioned by each paper and the proportion of the full list of principles mentioned by each paper. It should be noted that the percentages are based on the 58 principles summarized in step 1. Principles #46 and #57 were created after the matching process and therefore were not included here.

|  | Summarized principle | Horsky [1] | Bates [2] | Sittig [4] | Kuperman [19] | Phansalkar [20] | Pelayo [21] | Zachariah [22] | Horsky [23] | Payne [24] | Nb of papers |
| --- | --- | --- | --- | --- | --- | --- | --- | --- | --- | --- | --- |
| Improve the signal-to-noise ratio | #1 | X |  | X | X | X |  |  | X |  | 5 |
|  | #2 | X |  |  | X |  |  |  | X |  | 3 |
|  | #3 | X | X |  | X |  |  |  |  |  | 3 |
|  | #4 | X |  |  | X |  |  |  |  |  | 2 |
|  | #5 | X |  |  | X |  | X |  | X |  | 4 |
|  | #6 | X |  |  | X |  |  |  | X | X | 4 |
|  | #7 | X |  |  | X |  | X |  | X |  | 4 |
|  | #8 | X |  | X | X | X |  |  | X |  | 5 |
|  | #9 | X |  |  |  |  |  |  |  |  | 1 |
|  | #10 |  |  | X |  |  |  |  |  |  | 1 |
|  | #11 | X |  |  |  | X |  |  | X |  | 3 |
|  | #12 | X | X |  | X |  |  |  | X | X | 5 |
|  | #13 |  |  |  | X |  |  |  | X | X | 3 |
|  | #14 | X | X |  | X |  |  |  | X | X | 5 |
|  | #15 |  |  |  |  |  |  |  | X | X | 2 |
| Support collaborative work | #16 |  |  |  |  |  | X |  |  | X | 2 |
|  | #17 |  |  |  |  |  | X |  |  | X | 2 |
|  | #18 |  |  |  |  |  | X |  |  | X | 2 |
|  | #19 | X |  |  | X |  | X |  | X | X | 5 |
|  | #20 | X |  |  |  |  |  |  |  | X | 2 |
|  | #21 | X |  |  |  |  | X |  | X | X | 4 |
|  | #22 | X |  | X |  |  |  |  |  |  | 2 |
| Fit clinicians' workflow | #23 | X | X | X |  | X |  |  |  |  | 4 |
|  | #24 | X | X |  | X |  |  |  |  | X | 4 |
|  | #25 | X | X |  |  |  |  |  |  | X | 3 |
|  | #26 |  |  |  |  | X |  |  | X |  | 2 |
|  | #27 | X |  |  | X | X |  | X |  | X | 5 |
|  | #28 |  |  |  | X | X |  |  | X | X | 4 |
|  | #29 | X |  |  |  | X |  |  | X | X | 4 |
|  | #30 | X |  | X | X | X |  |  | X | X | 6 |
|  | #31 |  |  |  |  |  |  |  | X | X | 2 |
|  | #32 | X |  |  | X |  |  |  | X | X | 4 |
|  | #33 | X |  |  | X | X |  |  | X | X | 5 |
|  | #34 | X |  |  |  |  |  |  | X | X | 3 |
|  | #35 | X |  |  |  |  |  |  | X |  | 2 |
| Display relevant data | #36 | X |  |  |  | X |  |  | X | X | 4 |
|  | #37 | X |  |  | X | X |  |  | X | X | 5 |
|  | #38 |  |  |  | X | X |  |  | X | X | 4 |
|  | #39 | X |  |  |  | X |  |  | X | X | 4 |
|  | #40 |  |  |  |  |  |  |  | X | X | 2 |
|  | #41 | X |  | X |  |  |  |  | X | X | 4 |
|  | #42 | X | X |  | X | X | X | X | X | X | 8 |
|  | #43 | X | X |  | X |  |  |  | X | X | 5 |
| Make the system transparent | #44 |  |  |  | X |  |  |  |  | X | 2 |
|  | #45 | X |  |  |  | X |  |  | X |  | 3 |
|  | #47 |  |  |  |  | X |  |  |  | X | 2 |
|  | #48 |  |  |  |  | X |  |  |  |  | 1 |
| Include actionable tools | #49 | X | X | X | X | X | X | X | X | X | 9 |
|  | #50 | X |  |  | X |  |  | X | X | X | 5 |
|  | #51 |  |  |  |  |  |  | X | X | X | 3 |
|  | #52 | X |  |  |  |  |  | X | X | X | 4 |
|  | #53 | X |  |  |  |  |  |  |  | X | 2 |
|  | #54 |  |  |  |  |  |  |  |  | X | 1 |
|  | #55 |  |  |  |  |  |  |  |  | X | 1 |
|  | #56 |  |  |  |  |  |  |  |  | X | 1 |
|  | #58 |  |  |  | X |  |  | X | X | X | 4 |
|  | #59 | X |  |  |  |  |  |  | X |  | 2 |
|  | #60 | X |  |  | X |  |  |  |  |  | 2 |
| Nb of principles | | 39 | 9 | 8 | 27 | 19 | 9 | 7 | 37 | 40 |  |
| % | | 67,2 | 15,5 | 13,8 | 46,5 | 32,7 | 15,5 | 12,1 | 63,8 | 68,9 |  |
