# Supplementary material for: Evidence-based usability design principles for medication alerting systems
Source: BMC Med Inform Decis Mak. 2018 Jul 24;18:69. doi: 10.1186/s12911-018-0615-9 (PMC6057098; doi:10.1186/s12911-018-0615-9)
Supplement: Supplementary file 3 — Appendix 3. Results of the matching between instances of usability flaws (from Marcilly et al. [17]) and the usability design principles summarized in the present study. (DOCX 82 kb) [file 12911_2018_615_MOESM3_ESM.docx]

**Additional file 3: Appendix 3.** Results of the matching between instances of usability flaws (from Marcilly et al.[17]) and the usability design principles summarized in the present study.

| **Reference** | **Excerpts representing instances of usability flaws** | **Corresponding usability design principle(s)** | |
| --- | --- | --- | --- |
| [10;27] | "Repeated alerts within the same encounter or over multiple encounters for a given patient"; "alerts were sometimes excessively redundant. (...) The observer noted: we have now seen the same alert 4 times in the last 10 min or less." "Redundant alerts within a given patient encounter. Often triggered by renewing interacting medication pairs. E.g.,, Same alert appears a 3rd time” | #1 | #5 |
| [12;27] | "I see it does say "active" though. Technically, the [old] medication [order] isn't "active" because I just changed them to "discontinued" "Some meds should have been discontinued but were ‘active’, leading to extra alerts" | #2 |  |
| [10;27] | "Alert system does not distinguish between true allergies and bothersome, but non serious, side effects""System does not specifically target true allergies: Prescribers wanted alerts to distinguish between: allergy to exact medication versus drug class, serious reactions versus bothersome side effects" | #6 | #2 |
| [10;27] | "Alert conflicts with VAMC practices that are in place and/or standard medication practices." "Alert conflicts with common medication practices" | #13 |  |
| [10;27] | "Overuse of pop-ups, other, non-medication related pop-ups contribute to prescriber alert desensitization"; "Overuse of other, non-alert pop-up windows may contribute to desensitization" | #30 |  |
| [10;12;27] | "Multiple alerts were grouped together in one pop-up window." "Prescriber confusion when multiple alerts were presented [in the same screen]" "Multiple alerts presented in one pop-up" ""There are times when there are multiple flags [order checks] in the same box [pop-up alerts]"" | #31 |  |
| [10;12;27] | "Alert does not provide information on why it was triggered and/or the potential problem" "One physician said, “I wish it stated what the problem truly is, and simply. For example, simvastatin and diltiazem. [The alert] just says the drug names, not the problem."" "Alerts did not adequately indicate alert triggers, explain drug interactions, or describe the problem (e.g.,, “potentially causes hypotension”)" | #37 | #38 |
| [10;27] | "Alert does not provide clear information on relative risk of harm for a given patient" "Although some alerts are categorized by risk, (e.g.,, some are marked as “significant” and others are “critical”), this notation was not always sufficient for prescribers"; "Risk rating/degree of risk: Prescribers wanted a more clear indication of risk and suggested that alerts display a quantitative rating" [Inferred: there is no clear risk information in the alert] | #38 | #39 |
| [10;27] | "Alert is not evidence-based, does not provide a reference to evidence that does exist, and/or the actual or perceived level of evidence is low.""The alerts themselves do not present the evidence nor do they provide links to any supporting documentation" "Quality/strength of alert evidence; guidance on actions to take: Prescribers wanted alert to provide: references or links to evidence (...) some alerts were not evidence-based". "The actual or perceived level of evidence is low"; "unclear if the warnings were "evidence-based""; "extend of evidence is fair, stemming mainly from a few case reports" | #43 |  |
| [10;27] | "Alert system does not adequately reveal its capabilities/limitations to the prescriber; full functionality of the alert system is ambiguous." ; "System capabilities and limitations are ambiguous: Prescribers confused about whether the system could evaluate non-formulary, non-VA medications” | #44 |  |
| [8] | "There are too many [alerts]." | #1 |  |
| [8] | "Too many alerts or alerts at an inappropriate time: “Now we get alerts when we go to charting, which in my workflow is the last step. It’s after the patient’s gone. Now I get warned they’ve got some drug interaction. Great!” | #24 |  |
| [25] | "Most prescribers believed that most (alerts) were redundant." | #1 |  |
| [25] | "Most prescribers believed (…) that they received too many alerts …" | #1 |  |
| [25] | "Some doctors explained that the alerts appeared after they’d already made their prescribing decision and often provided them with information that they already knew." | #7 | #24 |
| [25] | "Every doctor reported that the alerts contained too much text and should be shortened." | #32 |  |
| [42] | “Decision support was mostly invisible” | #44 |  |
| [42] | "Inconsistent error prevention - able to prevent errors due to medication dose and frequency range, but not due to drug formulation (which is visible and changeable) or selection of incorrect patient or medication." | #44 |  |
| [37] | "The comments are merely stored in the database and displayed to no one, vital patient care instructions may be overlooked." | #19 |  |
| [37] | "All alerts include an acknowledgement comment field, only some alerts are marked as requiring acknowledgement. Even those alerts which require acknowledgement only require that the acknowledgement button be pressed, not that a comment be left, although clinicians may have misinterpreted the directive." | #58 |  |
| [9] | "Many [users] missed a question regarding data in the alert that should reduce the level of clinical concern (‘attenuating information’)" [rephrasing: this information is missed because of the organization of the information in the alert: it is outside the center of the alert] | #25 |  |
| [9] | "Some had difficulty identifying the patient’s risk factors for the interaction. Successfully answering this question required clicking on the Risk Factors tab to reveal the data (see Figure 3)." "The most commonly cited design concerns were the unnecessary use of tabs (7 subjects)" | #25 |  |
| [9] | "The most commonly cited design concerns were (…) lack of salience of the clinical effect (3 subjects)." | #33 |  |
| [9] | "The most common feature suggestions were (...) to provide more details about severity and clinical effect (3 subjects)." | #38 | #39 |
| [9] | "The most common feature suggestions were (…) to display whether a DDI is dose-dependent or idiosyncratic (4 subjects)…" | #40 |  |
| [9] | "The most common feature suggestions were to provide the normal ranges for labs (6 subjects)" [rephrasing: normal ranges for labs data are missing to interpret the results within the alerts] | #41 |  |
| [9] | "The most common feature suggestions were (…) to offer alternative treatment options (4 subjects)…" | #42 |  |
| [9] | "Two areas of minor criticism were (…) the lack of a more detailed reference section." | #43 |  |
| [30] | "Alerts (…) repeatedly encountered" | #1 |  |
| [30] | "Intrusive alerts presented at the wrong time in the workflow" | #24 |  |
| [30] | "Cost and health maintenance alerts that were intrusive to the workflow” | #30 |  |
| [30] | “Prescribers reported that alerts presenting during medication order entry were often (…) difficult to interpret in content and purpose” | #31 |  |
| [30] | "Prescribers reported that alerts presenting during medication order entry were often long (…) to interpret in content and purpose" " difficulty with (…) the length of the text." | #32 |  |
| [13] | "The reason for this is that the registration of an allergy is based on the ATC code, and the same drug can be registered under several codes if the drug has various indications." "The older version of the CPOE system only warns the user if the same drug code appears twice and not if the same drug (registered under different ATC codes) appears twice.” | #48 |  |
| [7] | "There is a reminder to add the corollary PTT check order. However, it is presented out of the logical workflow as an addition to the calculated dose alert and not at the end (Task 10), when the user reviews order completeness." | #24 |  |
| [7] | "The institutional guideline for heparin administration, however was embedded in the same alert containing the calculated dose, triggered later in the process when the planning stage of the order is generally completed" | #24 |  |
| [7] | "The institutional guideline for heparin administration (…) triggered later in the process when the planning stage of the order is generally completed" [inference: triggered too late] | #24 |  |
| [7] | "Three subjects expressed their need for better understanding of the dose calculation by the system. (...) users would not be able to "validate" the system's reasoning without resorting to calculation that is more complicated" [rephrasing: users are not sure that the system based its recommendation on the same assumptions they would have made] | #45 |  |
| [39] | "A major problem in Medicator is that the alert screen "medication dose units control" shows up too late in the ordering process" | #24 |  |
| [35] | "lack of patient tailored checking of medication order" | #6 |  |
| [35] | "Unclear information or guidance" in the messages | #31 |  |
| [35] | "Use of abbreviations and expressions that were not understandable by physicians, confusing terminology in labeling of buttons" | #35 |  |
| [35] | "erroneous system messaging [about how to solve the problem]" | #42 |  |
| [38] | "The CPOE system does not display information available on other hospital systems. For example, only the pharmacy’s computer provides drug interaction and lifetime limit warnings." | #20 |  |
| [38] | "CPOE provides feedback on drug allergies, but only after medications are ordered." | #24 |  |
| [26] | "excess alerts - e.g.,, asthma and opiate, warfarin and paracetamol" | #1 |  |
| [26] | "They also proposed some new alerts or eCDS functions (...) Patient-tailored threshold values, different from guideline-based" [rephrasing: alerts' thresholds are not patient-tailored] | #6 |  |
| [26] | ""Too low triggering threshold with drug interaction alerts"" | #8 |  |
| [26] | "Most of the drug alerts are not relevant for physiotherapists' works" | #20 |  |
| [26] | "Reminders do not support psychologists' work." | #20 |  |
| [26] | ""Mainly drug interaction alerts, and I [nurse] do not prescribe."" | #20 |  |
| [26] | "Reminders’ texts are sometimes too strict in the short version. If you don’t move the cursor over the text and see the whole reminder, the wording doesn’t work.’" | #25 |  |
| [26] | "Reminders' position on the left side of the screen" | #26 |  |
| [26] | ""Reminders’ texts are sometimes too strict in the short version. If you don’t move the cursor over the text and see the whole reminder, the wording doesn’t work."" |  |  |
| [31] | "Repetitive alerts are both annoying and unnecessary." | #1 |  |
| [31] | "they are "not right" for some reason." | #1 |  |
| [31] | "The system should distinguish between orders specified as "now" and those specified as "future" or "standing" and not consider them to be duplicates "It prompts you either way. I mean, you specifically made it standing or future."" | #5 |  |
| [31] | "They said that pop-up alerts particularly were annoying or unhelpful if they popped up "too early" in the encounter, or on the wrong screen." | #24 |  |
| [31] | "Alerts that are triggered by charting tasks rather than by ordering tasks may not be seen in the exam room workflow, as many clinicians complete their charting outside of the exam room, often after the patient has left." | #24 | #13 |
| [31] | "Yeah, you see it, but you have to do something to even find out what it means. "" [Inference: passive alerts are not sufficiently informational to support a quick triage] | #25 |  |
| [31] | "However, even some of these users acknowledged that "pop-up" alerts can be very annoying, ..." | #30 |  |
| [31] | "Some acknowledged they were unlikely to respond, or perhaps even be aware of alerts, unless they were intrusive." [rephrasing: alerts not sufficiently intrusive in this case] | #30 |  |
| [31] | "It was somewhat surprising that users did not always seem to understand how to use and manage the alerts effectively."[rephrasing: alert's management is not intuitive] | #46 |  |
| [31] | "There are instances where users expect the system to "be aware" of and utilize patient information that exists in the database." | #48 | #6 |
| [36] | "Reminders did not always apply given the context of a particular patient."; "The third barrier to following the advice of a clinical reminder was inapplicability to the specific situational context. For example, a recommendation to begin Highly Active Anti-Retroviral Therapy (HAART) was not followed because the patient had experienced multiple intolerances to the medication in the past." | #6 |  |
| [36] | "Ordering new medications satisfied the intent of the reminders but did not resolve them because they were not yet included in the logic." | #7 | #8 |
| [36] | "Reminders did not always match local practice" | #13 |  |
| [36] | "Users were uncertain how long the reminders would be turned off for each dialog option." | #46 |  |
| [36] | "In borderline cases, providers did not want to order medications yet but wanted to reconsider the action at the next visit." | #55 |  |
| [36] | "At all sites, at least one provider never satisfied reminders that were not clinically relevant, which required data entry such as when a patient received a vaccine at another hospital." | #58 |  |
| [33] | "When the physician used CPOE in the office area, a medication alert appeared saying ‘duplicate order’, which indicated that the patient was getting iron from both VA and non-VA sources (i.e., over-the-counter or non-VA pharmacy). Upon seeing this alert, the physician stated, “That’s not true to my knowledge. The patient doesn’t like to take it; I doubt he’s taking it [from a non-VA source].” | #2 |  |
| [12] | "VA's alert system did not match his/her (pharmacist) mental model of how an alert system should be designed (...) sometimes significant order checks really aren't significant (…) Now critical interactions (…) they are so many that are significant." | #8 | #23 |
| [33] | "The programmers' mental model, as reflected in the system image, did not adequately match prescribers' mental models (…): Physician (MD) orders [VA] aspirin - 162 mg. An order check [alert] appears. Says duplicate drug order. Non-VA ASPIRIN. [Alert] mentions 325mg....MD is looking at it also and [appears] confused" | #23 | #35 |
| [33] | "[Non VA system X] took up to 2-3 minutes to do the [medication] order check; (…) I haven't noticed much delay [with the VA system]" | #25 |  |
| [33] | "A specific medication alert did not appear, even though the pharmacist was expecting it to come up for a patient. (...) Pharmacist: "The patient is 85yrs old. It's not good if he is on a full dose of aspirin and Plavix. These are both anti-platelets [medications]..." [later, while ordering medications for this patient:] An order check appears [after the] pharmacist change[s] [the] aspirin dose. [Alert] says duplicate drug class - hydrocodone. This was the only order check alert listed in the window. Pharmacist: "This is because the patient is on hydrocodone. It pulled-up that one, but not the Plavix [alert] which is interesting."" "Programmer and prescriber mental model mismatches were evident when a prescriber expected an alert to appear, but the system did not display that alert: (…) physician [is] ordering [renewing] naproxen… No order check [alerts] appear." | #45 |  |
| [27] | "Low alert signal to noise ratio: numerousness of alerts" | #1 |  |
| [27] | "Some medication alerts may not be supported by pharmacy data. One pharmacist stated,“Sometimes, a doctor will call me about an interaction. I check in Micromedex®. [Micromedex® is a well-respected medication interaction database.10] Sometimes, there is no interaction shown in Micromedex® [for that alert]. I tell the doctor, ‘I don’t know. There is no information on it in the [Micromedex®] database.’” | #3 | #43 |
| [10] | "Number of alerts is problematic"; "Potential problems are overdetected or underdetected" | #1 |  |
| [10] | "Alerts sometimes inconsistent with EHR data: E.g.,, Alert: ‘metformin – no serum creatinine within 60 days’. NP: “This is often inaccurate.” | #2 |  |
| [10] | "Alert’s applicability to a certain patient/situation" "Alerts often valid, but not “applicable to the context”" | #6 |  |
| [10] | "Same alerts appear for a patient across one or more med renewals; E.g.,, Alert says ‘Previous adverse reaction to antidepressants.’…Phys types in override reason, ‘Has been on venlafaxine for 5 yrs now.’" | #6 | #59 |
| [10] | "Prescribers unsure if pharmacists review these (override justifications) or find them useful" | #19 | #16 |
| [10] | "Interface, which did not adequately support all prescriber types." | #21 |  |
| [10] | "Technology lags/down- times" "Even 10-15 second computer delays between the order and subsequent alert" | #25 |  |
| [10] | "Difficult to distinguish different alert types (e.g.,, duplicate drug versus duplicate drug class alerts)" | #29 |  |
| [10] | "Extraneous information decreases alert value: Prescribers wanted a brief description of the problem: e.g., "it needs to be 10 words or less"" | #32 |  |
| [10] | "(…) scrolling were problematic" [rephrasing: user needs to scroll to see the whole information] | #32 | #25 |
| [10] | "Salience: Alert visibility and distinction: Prescriber wanted more visual emphasis on high risk alerts" [rephrasing: no sufficient visual emphasis on high risk alerts] | #33 | #29 |
| [10] | "Language of alerts is difficult for prescriber to interpret: E.g.,, Alert says remote order checking unavailable. (See Figure 1.) Phys: “I do not know what that means.”" | #35 |  |
| [10] | "The alert did not provide essential patient information for the prescriber, even though it existed elsewhere in the EHR. For example, decision-making for some drug interaction alerts (e.g.,, amiloride and lisinopril, which can cause hyperkalemia) depend on patient labs (e.g.,, potassium). This missing, or more accurately, ‘hidden’ patient data triggered varying responses." | #41 |  |
| [10] | "guidance on actions to take" "Prescribers wanted alert to provide (…) advice on how to respond to the alert" | #42 |  |
| [10] | "Handbooks, Micromedex, and literature are consulted"; "Pharmacists are consulted real-time via phone or face-to-face; e.g.,, [Observer to NP: ‘What do you do if you are unfamiliar with an order check or have a question about it?’] NP: “Sometimes, I order the medication, because I know I can back out [cancel medication] after deeply looking into it. Sometimes, I do not order the medication. I get a book and look it up. If I don’t find it there, then I call the clinical pharmacist.” " [rephrasing: the alert content lacks of information] | #43 |  |
| [10] | Prescribers unaware that they could turn off some alerts" | #46 |  |
| [10] | "Ambiguity about alert management; need for closed-loop feedback; E.g.,, Phys: “I want [the alert system] to have user control. I am not confident it’s checking all the interactions that I want it to check.”" | #48 |  |
| [10] | "No alerts for free text medications" "Free-text entry (e.g.,, ‘OK’) may not be effective (in the override justification logic)" | #48 | #58 |
| [10] | "Alerts cannot be pulled up later, as needed, hindering alert resolution" ; "Sometimes, prescribers wanted a way to retrieve an alert that had been displayed, but the alert system did not support this function." | #55 |  |
| [10] | "Time needed to resolve alerts: Justification requirement often viewed as time burden" [rephrasing: justify the irrelevance of an alert requires several actions that take time] | #58 |  |
| [10] | "System capabilities and limitations are ambiguous" |  |  |
| [40] | "In the current design, this data [summary of patient information] is only visible by switching tabs when completing CRs." | #25 |  |
| [40] | "Two participants misinterpreted the meaning of “When” to represent the last time the current patient received the intervention instead of the frequency the intervention is due for all patients." | #34 |  |
| [28] | "Clinicians (…) faced with a long list of them for each patient." | #1 |  |
| [28] | "The CRs appear on this cover sheet but are delayed in loading and displaying (Site 3 reported an average delay of 8 seconds for the CRs to load)." | #25 |  |
| [28] | "When defaulting past the cover sheet, feedback for the presence of due CRs is signified by a question mark icon in the upper right corner of the display. We observed three providers misinterpret this question mark to indicate that the patient had no CRs due, when in actuality it meant the system was still evaluating data to determine which CRs were due." | #34 |  |
| [28] | "There were cases in which clinicians reached a dead end within the CR system, with no reasonable option to proceed." "We observed instances in which none of the available options to satisfy the CR applied to the patient or situation." | #49 |  |
| [28] | "Clinicians reported that they faced situations in which CRs could not be removed and therefore continued to appear." | #59 |  |
| [28] | "Five nurses and two providers were observed to skip all or some of the reminders and explained that this was because they perceived that they did not have enough time to ‘‘satisfy’’ the reminders by entering data.”; "Completing the CRs creates ‘‘double documentation’’ burdens for some providers (…) as they generally keep track of this information without the CRs (e.g.,, in a health maintenance list within the progress note)."[Inference: satisfy the reminders requires time] | #60 |  |
| [28] | "CR system is currently insufficient for supporting transmission of reminder results from nursing intake to provider examination" | #56 |  |
| [28] | "The use of CRs automatically generates text that is added to the progress note, but that text is not integrated with the template information and is generally added to the bottom of the note." | #57 |  |
| [28] | "Options within the dialogue box of the CRs do not always match the patient’s response or there is not an appropriate option for indicating why the provider has decided not to order a test, for example." | #58 |  |
| [41] | "“It is hard to use the tool when sitting with a patient because it is in paragraph form. It would be better if factoids or outlines and standardized approaches are numbered or outlined and in lists.”" | #33 | #31 |
| [41] | “The page is too convoluted. When there are 10 different things on the screen, providers aren’t going to read any of it.” | #33 | #32 |
| [41] | "Several users in the simulation-based testing did not notice the arrows under the clinical recommendations or did not realize they provided additional, more detailed information about the basic recommendation when clicked on." | #34 |  |
| [41] | "A user thought that the appearance of the “stamp” window implied that the patient had a chronic pain problem or diagnosis. In actuality, the “stamp” indicated that the patient had a scheduled appointment within a 5-day window and that ATHENA-OT had recommendations available should the provider consider OT for that patient." | #34 |  |
| [41] | "participants commonly made specific clinical suggestions, such as clarifying the wording of recommendations, cautions, and data table notation" [rephrasing: it is not clear enough] | #35 |  |
| [41] | "They wanted the CDSS to tell them whether to initiate or discontinue therapy or increase or decrease dosing rather than provide a detailed explanation of how to evaluate these potential actions." | #42 |  |
| [41] | "Another in-clinic user stated that the system would be more helpful if it gave more detailed information on “how to switch or discontinue drugs.”" | #50 | #51 |
| [41] | "I would like more of the recommendations to go into a note to document what I have done with the patient" | #57 |  |
| [29] | "The quantity or number of alerts (generated or overridden) is mentioned [as a reason to turn off an alert]" [Inferred: there are too many alerts] | #1 |  |
| [29] | "The respondents mention that the alert does not need any action or that they never perform any action [as a reason to turn off an alert]" [Inferred: the alert is useless since no action has to be taken] | #1 |  |
| [29] | "The rapidity of the adverse effect is mentioned [as a reason to turn off an alert]" [Inferred: problem of relevance of the alert] | #5 |  |
| [29] | "The fact that effects are monitored or serum level measured is mentioned [as a reason to turn off an alert]" [Inferred: monitoring: the alert is triggered while the corrective actions are already taken] | #7 |  |
| [29] | "The fact that only specialists are prescribing a specific drug or the combination of drugs is mentioned [as a reason to turn off an alert]" [Inferred: alert is inconsistent with clinical practice] "The fact that the drugs are intentionally combined because of a desired effect of the DDI or the fact that they are generally combined for other reasons is mentioned [as a reason to turn off an alert]" [Inferred: alert is inconsistent with clinical practice] | #7 |  |
| [29] | "The fact that the alert is know is mentioned [as a reason to turn off an alert]" [inference: alert is redundant] | #7 |  |
| [29] | "The incidence of adverse events due to the DDI is mentioned [as a reason to turn off an alert]" [Inferred: Irrelevance of the alerts due to their incidence] | #8 |  |
| [29] | "DDI is mentioned to be (…) not serious [as a reason to turn off an alert]" [Inferred: the alert is not sufficiently relevant] | #8 |  |
| [29] | "One surgical resident did not understand the text of the sequence-dependent alerts well, considered the administration of drugs was out of the control of physicians, and thought these alerts therefore irrelevant" | #20 |  |
| [29] | "[missing] information in the alert text (…) (recommendations to adjust doses, to measure serum levels, monitor patient parameters, or prescribe alternative drugs) | #42 |  |
| [29] | "[missing] evidence of the DDI (…) [as a reason to turn off an alert]" [Inferred: there is no evidence mentioned in the alert, or not sufficiently serious] | #43 |  |
| [11] | "Too low dose limits" | #1 |  |
| [11] | There are so many drug–drug interactions that are irrelevant, that I am often inclined to rapidly click them away [resident in internal medicine]. | #1 |  |
| [11] | “DDIs that should be suppressed because of low incidence of adverse events" | #8 |  |
| [11] | "Alerts regarding drug administration times should be handled by nurses [while they are displayed to physicians]" | #20 |  |
| [11] | "Necessity for scrolling down the whole alert text to find the conclusion" | #25 | #31 |
| [11] | ""You do not get the warning again, and there is no button to get it [the second alert] back"" | #55 |  |
| [11] | “I did not look through the new screen, and then I hit the button and suddenly [the alert] was gone” | #55 |  |
| [32] | "According to clinicians, the sensitivity of alerts was often set too high while the specificity was too low." | #1 |  |
| [32] | "Within-class interactions typically reflect an out-of-date medication list – such as an antibiotic interacting with another antibiotic – rather than a true interaction." | #2 |  |
| [32] | "Pregnancy alerts would be more useful (…) if they were suppressed for male patients and women of non-child-bearing age." | #6 |  |
| [32] | "Appropriate polypharmacy is not acknowledged" "In psychiatric care, mood stabilizers are often used intentionally in combination or “augmentation” therapy with antidepressants." | #7 |  |
| [32] | "Since most pediatric drugs are used off-label, pediatricians find it difficult to interpret the validity of many alerts." | #7 |  |
| [32] | "Alerts are often fired for drug combinations that conform to clinical guidelines and are recommended by specialist colleagues. Examples include aspirin with angiotensin converting enzyme inhibitors in patients with heart disease or diabetes." | #7 |  |
| [32] | "There was insufficient discrimination between alerts of varying severity." | #29 |  |
| [32] | "Pregnancy alerts would be more useful if they showed the category of alert (A, B, C, etc.) rather than narrative information" | #29 |  |
| [34] | "Alerts once entered in the system can be outdated. The processes how to keep them up-to-date is not yet implemented (i.e., for patients who were carrier of methicillin-resistant staphylococcus aureus (MRSA) and who are now readmitted to the hospital)." | #2 |  |
| [34] | "Reminder alerts that should be given the last day of hospitalization (i.e., bacteriological tests), a day the system cannot forecast. This leads to an alert every day.” | #6 |  |
| [34] | "Some express doubts on whether the system has up-to-date information (for instance for weight-based drug dosage alerts in pediatrics or drug interactions in cardiology where they often introduce new drugs). | #13 | #3 |
| [34] | "Some alerts are out of the visual focus region when using the system" | #26 |  |
| [34] | "Visualization of drug-drug interaction alerts where one drug has interactions with several others" [Inference: no sufficiently structured presentation] | #31 |  |
| [22] | "Alerts are not stratified by levels of severity" | #8 | #28 |
| [22] | "alerts are not grouped according to severity" | #11 |  |
| [22] | "The user must scroll through the screen to see all alerts" | #25 |  |
| [22] | "All alerts are labeled with ‘Warning'" | #28 |  |
| [22] | "No shapes or icons are used to convey alert priority." (System 3) | #29 |  |
| [22] | "Alerts are color-coded by severity, not by the type of alert" (System 1) | #29 |  |
| [22] | "Alerts are color-coded by severity, not by type" (System 3) | #29 |  |
| [22] | "Color coding within the alert is not utilized in any way" | #29 |  |
| [22] | "Neither color, signal words, nor shapes or icons are used to signify the alert’s priority; all alerts possess the same visual characteristics and appear as drugs orders are placed" (System 2) | #29 |  |
| [22] | "The user can link to outside sources of information from elsewhere in the system when connected to a workstation, but there is no link within the alert" | #31 | #41 |
| [22] | "All alerts only present information, and no alerts require action" | #42 |  |
| [22] | "No catalog exists explaining the levels of alert severity" (system 3) | #47 |  |
| [22] | "No catalog is visible for the user that explains in detail the levels of severity" (System 1) | #47 |  |
| [22] | "Directly from the alert, the user can choose to continue with or cancel the order of the offending drug; no other actionable options are available." | #49 |  |
